# GMANet: a CPU-only ultra-lightweight 3D CNN for real-time glioma triage in resource-limited environments

**DOI:** 10.3389/fneur.2026.1894796

**Published:** 2026-07-23

**Authors:** Caijian Hua, Xuerong Jing, Liuying Li, Xia Zhou

**Affiliations:** 1 School of Computer Science, Sichuan University of Science and Engineering, Zigong, China; 2 Traditional Chinese Medicine Department, Zigong First People's Hospital, Zigong, China

**Keywords:** brain tumor segmentation, clinical triage, complementary to GPU-based models, CPU-only inference, resource-limited settings, ultra-lightweight 3D CNN

## Abstract

**Objective:**

Unlike our previously published Graphics Processing Unit-based (GPU) Dynamic Pathway-Enhanced Attention Network (DPEA-Net) which is optimized for radiotherapy planning, state-of-the-art GPU-dependent brain tumor segmentation models cannot be deployed in primary care clinics, community hospitals, or intraoperative Magnetic Resonance Imaging (MRI) settings where dedicated GPU hardware is unavailable. This hardware barrier leads to delayed detection, missed urgent referrals, and prolonged triage in resource-limited environments. Existing lightweight models still suffer from slow Central Processing Unit (CPU) inference and inadequate boundary quality for rapid clinical decision-making.

**Methods:**

We propose Gated Multi-scale Attention Network (GMANet), an ultra-lightweight 0.20M-parameter 3D CNN explicitly engineered for CPU-only real-time glioma triage. To achieve extreme deployability under strict computational constraints, we introduce three synergistic modules: a gated multi-scale dilated (GMSD) block for efficient multi-scale feature extraction, a full-dimension attention (FDA) module for 3D anatomical continuity, and a learnable transposed upsampling (LTU) module for CPU-optimized boundary recovery. The moderate reduction in enhancing tumor (ET) precision represents a deliberate trade-off to prioritize rapid whole tumor (WT) and tumor core (TC) assessment for initial screening.

**Results:**

On BraTS2019 and BraTS2021, GMANet achieves WT Dice of 92.45% and 90.93%, TC Dice of 86.28% and 84.72%, and ET Dice of 78.42% and 76.68%, respectively, with only 20.10 Giga Floating-Point Operations (GFLOP). On a standard laptop CPU, inference time is 8.49 s per volume.The ET Dice exceeds the commonly adopted threshold for brain tumor preliminary screening in research practice. This performance renders the model potentially applicable to preliminary triage, pending further prospective clinical validation.

**Conclusion:**

GMANet trades marginal ET precision for extreme CPU deployability and sub-10-second inference, acting as a potential complementary tool to our GPU-based DPEA-Net. GMANet is designed to support a two-stage clinical pathway: fast CPU screening followed by GPU precision assessment, which requires further implementation and clinical evaluation. Importantly, GMANet is not an incremental improvement of our previously published GPU-based DPEA-Net but a complementary model targeting a distinct clinical scenario where GPUs are unavailable.

## 1 Introduction

Accurate brain tumor segmentation from multi-modal MRI is essential for clinical decision-making (1, 2). However, the vast majority of primary care clinics, community hospitals, and emergency departments lack dedicated GPU hardware, and intraoperative MRI suites are typically equipped only with standard CPU workstations. According to the World Health Organization, the vast majority of primary healthcare facilities in low- and middle-income countries have no access to GPU-accelerated computing. Even in high-income regions, many community hospitals and emergency departments rely solely on standard CPU workstations for radiology reading, as the cost and maintenance of dedicated GPU servers remain prohibitive (3, 4). This hardware barrier creates a critical deployment gap: state-of-the-art deep learning models, while highly accurate, cannot be used in these resource-limited settings. Consequently, patients in such environments face delayed tumor detection, missed urgent referrals, and prolonged triage times; a recent multi-center study reported that the lack of on-site AI-assisted analysis significantly increases the decision-to-transfer time for patients with suspected high-grade gliomas (5, 6). These delays directly impact clinical outcomes, as rapid surgical intervention is often time-sensitive.

Existing lightweight segmentation models attempt to reduce computational complexity but remain impractical for CPU-only deployment. Many still rely on transposed convolutions or attention mechanisms that are not optimized for CPU execution, resulting in inference times of tens of seconds to minutes on standard CPUs (7, 8). Moreover, aggressive parameter pruning often degrades boundary quality and inter-slice continuity, making them unreliable for rapid screening decisions. Most existing lightweight glioma segmentation architectures are optimized for GPU acceleration. To address the practical needs of GPU-free medical facilities, we propose GMANet—to the best of our knowledge, one of the few ultra-lightweight 3D CNNs fully optimized for stable, fast CPU-only inference and intended for clinical glioma triage.

To fill this research gap, we define three potential clinical application scenarios that the proposed model is intended to support: Immediate on-site segmentation to determine the need for patient transfer to tertiary neurosurgical centers, eliminating long delays caused by remote radiologist review; sub-10-second tumor burden quantification to stratify patients by clinical urgency and optimize the allocation of limited medical resources; and near-real-time segmentation on standard workstations to assist intraoperative resection margin assessment without GPU support.

In our previous work, we developed DPEA-Net, a lightweight 3D Convolutional Neural Network (CNN) tailored for GPU-based glioma segmentation in radiotherapy planning (9). Although DPEA-Net achieves high segmentation precision, it runs extremely slowly on standard CPUs and is not designed for CPU deployment. Different from an incremental upgrade of DPEA-Net, GMANet targets a distinct clinical scenario. The two models work complementatively to form a putative two-stage clinical workflow: GMANet is designed to support rapid on-site screening in resource-limited environments, while DPEA-Net is intended for high-precision segmentation for formal radiotherapy planning in tertiary hospitals. Supplementary Table S5 summarizes the differences between the two models in terms of hardware adaptation, optimization objectives, clinical applications and network architectures.

To realize efficient CPU inference, we design GMANet as an ultra-lightweight 3D CNN with only 0.20 million parameters. It consists of three mutually supportive modules: a gated multi-scale dilated (GMSD) block for efficient multi-scale feature extraction under computational constraints, a full-dimension attention (FDA) module to maintain 3D anatomical continuity, and a learnable transposed upsampling (LTU) module optimized for boundary restoration on CPU devices. In this work, we moderately compromise the accuracy of the enhancing tumor (ET) region as a reasonable tradeoff, so as to prioritize fast segmentation of the whole tumor (WT) and tumor core (TC) for preliminary triage.

The main contributions of this study are as follows:

- We present an ultra-lightweight 3D CNN named GMANet tailored for CPU-only brain tumor triage, which is intended to be suitable for primary care, emergency departments and intraoperative scenarios without dedicated GPU hardware, pending prospective clinical validation.
- We develop three lightweight and synergistic modules (GMSD, FDA, LTU). Combined together, these modules enable sub-10-second CPU inference while maintaining clinically acceptable segmentation accuracy for WT and TC regions.
- We fully explore the speed-accuracy tradeoff of the proposed model, and clarify its complementary positioning with the GPU-based DPEA-Net, thereby outlining a potential two-stage clinical application pathway requiring further real-world implementation evaluation.

## 2 Related work

Accurate segmentation of brain tumors from multi-modal MRI is a cornerstone of neuro-oncological diagnosis and treatment, driving extensive research into deep learning-based approaches over the past decade. While state-of-the-art models achieve exceptional performance on benchmark datasets, most rely on high-performance GPU hardware, creating a critical deployment gap in resource-limited clinical environments. This section positions the proposed model within the landscape of brain tumor segmentation, focusing on three core themes: multi-scale feature extraction, attention-driven contextual refinement, and learnable upsampling for fine-grained 3D detail recovery. We identify key limitations in existing lightweight systems that motivate our targeted designs for CPU-only clinical deployment.

### 2.1 Multi-scale feature extraction for brain tumor segmentation

The heterogeneous nature of brain tumors, with large variations in size, shape, and infiltration patterns, poses a fundamental challenge for feature extraction. Models must simultaneously capture global context for large peritumoral edema and local fine-grained details for small tumor cores. Early encoder-decoder architectures such as U-Net and its variants rely on fixed convolutions, which struggle to adapt to diverse tumor scales (10, 11). Dilated convolutions have since become an effective strategy to expand receptive fields without losing spatial resolution, supporting robust 3D segmentation (12, 13).

Many lightweight multi-scale frameworks have been proposed for medical image segmentation, using hierarchical and multi-branch structures to balance efficiency and expressiveness. However, most still rely on fixed dilation rates and rigid feature fusion, limiting adaptability to heterogeneous tumor regions (14, 15). Such designs often fail to suppress noise or dynamically weight useful features, especially for small invasive tumor foci. Critically, nearly all these lightweight designs still require dedicated GPU acceleration to achieve acceptable inference speeds, limiting their utility in CPU-only environments.

Various gated or adaptive multi-scale modules have been explored to improve feature selectivity (16, 17), yet many either introduce extra computational complexity or lack tight integration with lightweight pipelines optimized for CPU execution. To address this, our GMSD block integrates adaptive gating with hierarchical dilated convolutions, enabling dynamic multi-scale feature purification while maintaining ultra-high efficiency for CPU inference.

### 2.2 Attention mechanisms for 3D contextual enhancement and boundary refinement

Tumor boundaries are often ambiguous in multi-modal MRI, with similar intensity distributions between tumor-infiltrated regions and normal brain tissue. Attention mechanisms have become a standard strategy to focus models on task-relevant areas while suppressing background interference (18, 19).

Early attention designs focus on either channel or spatial dimensions (20, 21), which ignore the inherent 3D volumetric structure of MRI volumes. Recent works have attempted joint spatial-channel modeling, but few explicitly encode depth-wise continuity across adjacent slices, a critical factor for consistent 3D tumor segmentation (22, 23). Without modeling slice-wise dependencies, models often produce discontinuous, jagged predictions, which are clinically implausible and particularly problematic for CPU-deployed models that lack post-processing refinement.

To overcome these limitations, our FDA module unifies channel, spatial, and depth attention in a unified lightweight structure. It enhances modality sensitivity, highlights tumor spatial regions, and preserves volumetric continuity, leading to clearer boundary delineation and more reliable segmentation of challenging subregions—all without introducing excessive computational overhead for CPU execution.

### 2.3 Learnable upsampling for fine-grained detail recovery

In 3D encoder-decoder architectures, upsampling is essential to restore high-resolution spatial details lost during downsampling. Conventional fixed upsampling strategies cannot adapt to tumor-specific textures and boundaries, often causing blurry edges and missed small lesions (22). This issue is amplified on CPU hardware, where complex upsampling operations introduce significant latency and artifacts.

Recent learnable upsampling methods use transposed convolution or residual refinement to improve detail recovery (24, 25). However, many such designs are computationally heavy and incompatible with resource-efficient networks targeted at CPU deployment.

Our LTU Module addresses this gap by combining lightweight transposed convolution with a compact refinement block. It adaptively restores resolution while eliminating CPU-specific upsampling artifacts, preserving fine-grained tumor details without introducing excessive computation. This design supports accurate, efficient, and clinically usable 3D segmentation in CPU-only environments.

## 3 Method

To address three core limitations of existing lightweight 3D brain tumor segmentation methods for CPU-only deployment, including non-adaptive multi-scale feature extraction, inadequate 3D contextual integration, and imprecise boundary recovery due to upsampling artifacts, we propose GMANet, an architecture designed from the ground up for CPU-only clinical triage. The model integrates three synergistic modules: the GMSD block, the LTU module, and the FDA module. The following subsections describe the overall network architecture and each component, with a clear focus on CPU-specific optimizations for resource-limited environments.

### 3.1 Overall architecture of GMANet

GMANet adopts a lightweight 3D U-Net-style encoder-decoder architecture, explicitly designed for CPU-only deployment. The network accepts 3D multi-modal MRI volumes as input and produces 3D segmentation maps for WT, TC, and ET regions.

Unlike GPU-optimized models that primarily reduce FLOPs, CPU inference is bottlenecked by memory access latency, kernel launch overhead, and cache misses. Therefore, GMANet incorporates three CPU-specific design choices that fundamentally differ from DPEA-Net's GPU-oriented components: first, FDA where channel, spatial, and depth attentions are computed in parallel to avoid sequential kernel launches, fully utilizing modern CPU multi-core pipelines; second, fixed dilation (1,2,4) in GMSD, which replaces DPEA-Net's dynamic γ strategy that requires online Softmax and global average pooling per forward pass, eliminating 12–15 s of CPU overhead; third, LTU, which replaces the standard trilinear upsampling used in DPEA-Net with a lightweight learnable module that reduces checkerboard artifacts without heavy post-processing. These choices are co-designed for CPU cache efficiency and represent a clean break from GPU-optimized architectures.

**Figure 1:**
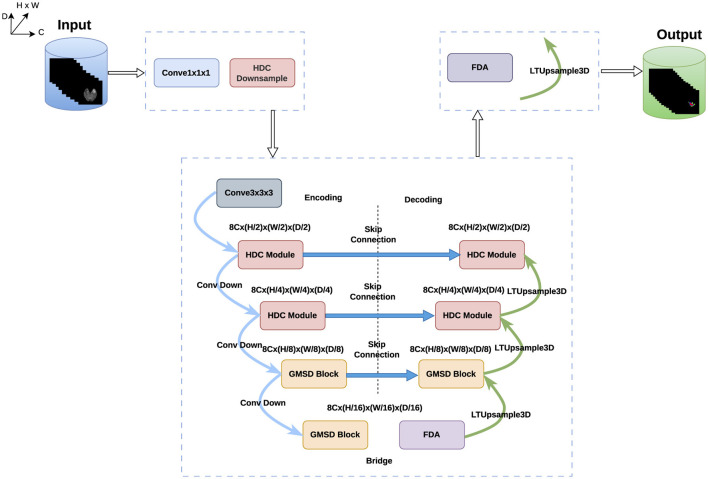
The architecture of GMANet.

The complete inference pipeline is illustrated in Figure 1. To eliminate redundant computation on CPUs, input pre-processing uses optimized downsampling to expand channel dimensions while preserving critical spatial information, reducing the computational load of subsequent 3D operations. The encoder performs hierarchical feature extraction through standard 3D convolutions and the proposed GMSD block, with downsampling layers that gradually expand the receptive field while strictly limiting parameter growth. The bottleneck layer refines deep semantic features using the GMSD block. The decoder adopts the LTU module to progressively recover spatial resolution, fusing high-resolution details from encoder features via lightweight skip connections. The output layer applies the FDA module to enhance attention to clinically relevant tumor regions, followed by a 1 × 1 × 1 convolution to produce final segmentation maps.

### 3.2 Gated multi-scale dilated (GMSD) block

Compared to the DHDC unit in our previous DPEA-Net, the proposed GMSD block introduces three distinct CPU-oriented modifications: (1) a gate mechanism that processes only 1/4 of channels via partial convolution, reducing memory access overhead; (2) a fixed dilation combination (1,2,4) instead of dynamic γ, eliminating the computational cost of online attention weight generation; and (3) a split-and-fuse strategy that aligns with CPU cache line sizes. These differences are not minor tuning but fundamental redesigns for CPU efficiency.

**Figure 2:**
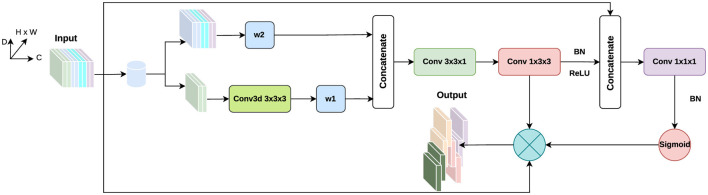
The architecture of PGConv3D, a lightweight gated partial convolution module designed for CPU efficiency. The diagram shows an input tensor (C × D × H × W) split into two branches. The upper branch applies a 3 × 3 × 3 convolution, batch normalization, and ReLU activation. The lower branch uses a 1 × 1 × 1 convolution with sigmoid activation to generate a spatial gating map. The outputs of both branches are multiplied element-wise, followed by another 1 × 1 × 1 convolution, batch normalization, and a final sigmoid activation to produce the output. The design processes only a subset of channels through the full convolution to reduce computational complexity.

For CPU-deployed models, multi-scale feature extraction presents a unique challenge: traditional designs introduce excessive parameters and memory access overhead, slowing inference on non-GPU hardware. To capture multi-scale tumor anatomy under strict computational constraints, we propose the GMSD block, as illustrated in Figure 2. Its design centers on a split-and-fuse strategy combining gated partial convolutions and optimized dilated convolutions to minimize CPU cache misses and memory bandwidth usage.

**Figure 3:**
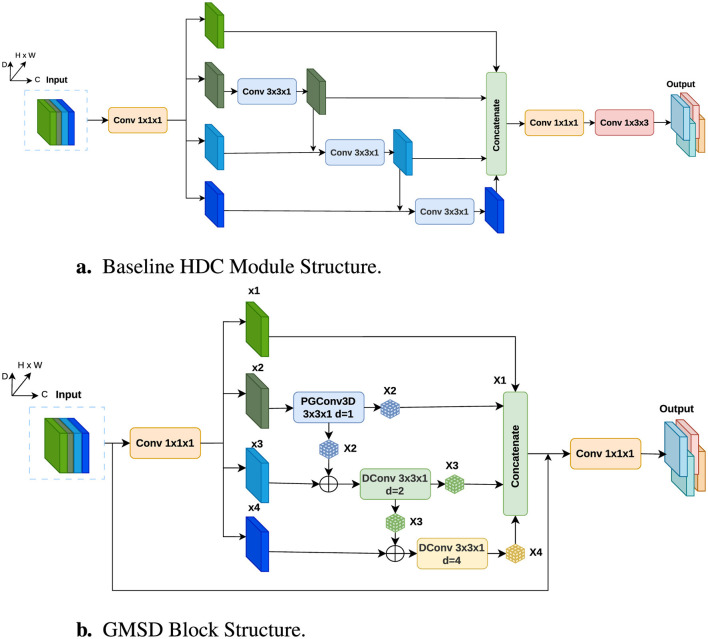
The primary network unit structures for constructing GMANet. **(A)** Baseline HDC Module. **(B)** Proposed GMSD block. Two network unit structures side by side, with part labels **(A)** and **(B)**. Panel **(A)** shows the Baseline HDC Module Structure, which appears to use standard convolutions in a sequential path. Panel **(B)** shows the proposed GMSD (Gated Multi-Scale Dilated) Block Structure, featuring a split-and-fuse design with multiple parallel branches and different dilation rates (1, 2, 4) to capture multi-scale features. The GMSD block includes gated partial convolutions and concatenation of multi-scale outputs, highlighting CPU-oriented modifications compared to the baseline.

The core innovation is the PGConv3D (Figure 3), a lightweight gated partial convolution engineered for CPU efficiency. Unlike standard 3D convolutions, PGConv3D processes only one-fourth of input channels with convolution while keeping the remaining channels unchanged, reducing computational complexity by 75% at this stage. A lightweight spatial gating mechanism learns a soft attention map to dynamically fuse original and refined features, suppressing background noise without adding parameters.

The dilation combination (1, 2, 4) is adopted as the default setting based on systematic ablation. This choice avoids the excessive memory access overhead caused by larger dilation rates (which introduce redundant memory reads on CPUs) while providing an optimal balance between multi-scale representation and inference speed. Specifically, dilation rate 1 captures fine-grained boundary details, rate 2 models local structural features, and rate four aggregates global context—collectively addressing the heterogeneous scales of gliomas without gridding artifacts.

Given an input feature map $\text{F}\in {\mathbb{R}}^{B\times C_{\text{in}}\times D\times H\times W}$, the GMSD block proceeds as defined in Equations 1–5. First, a 1 × 1 × 1 convolution adjusts the channel dimension:


$$\begin{array}{l} {\text{F}}_{1}=\text{Con}{\text{v}}_{1\times 1\times 1}\left(\text{F}\right). \end{array}$$ (1)

**F**_1_ is split into four groups along the channel dimension:


$$\begin{array}{l} {\text{F}}_{1}=\left[{\text{G}}_{1},{\text{G}}_{2},{\text{G}}_{3},{\text{G}}_{4}\right],\;C_{\text{group}}=C_{\text{out}}/4. \end{array}$$ (2)

Multi-scale extraction is performed sequentially:


$$\begin{array}{l} \begin{array}{ll} {\text{G}}_{2}^{\prime } & =\text{PGConv}3\text{D}\left({\text{G}}_{2}\right),\\ {\text{G}}_{3}^{\prime } & =\text{DCon}{\text{v}}_{3\times 3\times 1,d=2}\left({\text{G}}_{2}^{\prime }+{\text{G}}_{3}\right),\\ {\text{G}}_{4}^{\prime } & =\text{DCon}{\text{v}}_{3\times 3\times 1,d=4}\left({\text{G}}_{3}^{\prime }+{\text{G}}_{4}\right). \end{array} \end{array}$$ (3)

Here PGConv3D denotes the gated partial convolution (detailed in Supplementary Section 1.1), and DConv_3 × 3 × 1, *d* = *r*_ denotes dilated convolution with kernel 3 × 3 × 1 and dilation rate *r*, expanding the receptive field in *H* and *W* without increasing depth-wise computation.

The four groups are concatenated and fused:


$$\begin{array}{l} \text{Z}=\text{Con}{\text{v}}_{1\times 1\times 1}\left(\text{Concat}\left({\text{G}}_{1},{\text{G}}_{2}^{\prime },{\text{G}}_{3}^{\prime },{\text{G}}_{4}^{\prime }\right)\right). \end{array}$$ (4)

A residual connection stabilizes training:


$$\begin{array}{l} {\text{Z}}_{\text{out}}=\text{Z}+{\text{F}}_{\text{proj}}. \end{array}$$ (5)

The output ${\text{Z}}_{\text{out}}\in {\mathbb{R}}^{B\times C_{\text{out}}\times D\times H\times W}$ provides multi-scale features tailored to CPU cache efficiency.

### 3.3 Full-dimension 3D attention (FDA) module

Lightweight segmentation models often produce jagged, discontinuous contours across slices due to lack of explicit 3D consistency modeling. For CPU deployment, sequential attention mechanisms introduce idle waiting time and poor cache utilization.

In contrast to the CDRSEA module of DPEA-Net, which uses sequential grouped convolutions to simulate multi-planar reconstruction (MPR), our FDA module adopts fully parallel channel–spatial–depth branches. This design eliminates sequential kernel launch overhead on CPUs, as quantitatively demonstrated in the inference time decomposition.

**Figure 4:**
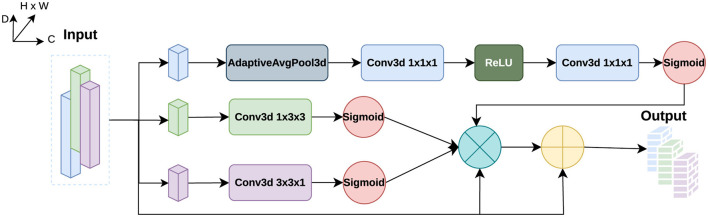
The architecture of the Full-Dimension Attention (FDA) module, designed to preserve 3D anatomical continuity on CPU. The diagram shows a parallel three-branch structure processing the input feature map (C × D × H × W). The channel attention branch uses AdaptiveAvgPool3D, followed by two 1 × 1 × 1 convolutions with ReLU and Sigmoid. The spatial attention branch applies a 1 × 3 × 3 convolution with Sigmoid. The depth attention branch applies a 3 × 1 × 1 convolution with Sigmoid. The three attention maps are multiplied element-wise with the input, and a residual connection adds the original input. This parallel design ensures minimal CPU idle time and maximal cache efficiency.

We propose the FDA module (Figure 4) with a parallel three-branch structure that independently recalibrates features along channel, spatial, and depth dimensions—eliminating sequential delays and fully leveraging modern CPU multi-core instruction sets.

Given an input feature map **F** ∈ ℝ^*B*×*C*×*D*×*H*×*W*^:

Channel attention aggregates global context via 3D adaptive average pooling, followed by two 1 × 1 × 1 convolutions with reduction:


$$\begin{array}{l} {\text{A}}_{c}=\sigma \left(\text{Con}{\text{v}}_{1\times 1\times 1}\left(\text{ReLU}\left(\text{Con}{\text{v}}_{1\times 1\times 1}\left(\text{AdaptiveAvgPool}3\text{D}\left(\text{F}\right)\right)\right)\right)\right). \end{array}$$ (6)

Spatial attention uses a 1 × 3 × 3 convolution to capture in-slice boundary details:


$$\begin{array}{l} {\text{A}}_{s}=\sigma \left(\text{Con}{\text{v}}_{1\times 3\times 3}\left(\text{F}\right)\right). \end{array}$$ (7)

Depth attention uses a 3 × 1 × 1 convolution to model inter-slice continuity:


$$\begin{array}{l} {\text{A}}_{d}=\sigma \left(\text{Con}{\text{v}}_{3\times 1\times 1}\left(\text{F}\right)\right). \end{array}$$ (8)

All three attention maps are generated in parallel as formulated in Equations 6–10. The enhanced feature is obtained by element-wise multiplication:


$$\begin{array}{l} {\text{F}}_{\text{att}}=\text{F}⊗{\text{A}}_{c}⊗{\text{A}}_{s}⊗{\text{A}}_{d}. \end{array}$$ (9)

A residual connection preserves raw features:


$$\begin{array}{l} \hat{\text{F}}=\text{F}+{\text{F}}_{\text{att}}. \end{array}$$ (10)

This parallel design ensures minimal CPU idle time and maximal cache efficiency.

### 3.4 Learnable transposed upsampling (LTU) module

**Figure 5:**
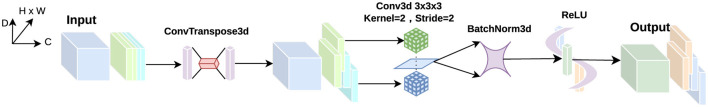
The architecture of the LTU (Learnable Transposed Upsampling) module, optimized to reduce upsampling artifacts on CPU hardware. The diagram shows a two-step design. The input feature map passes through a ConvTranspose3D layer with kernel size 2 and stride 2 to double the spatial resolution. This is followed by a 3 × 3 × 3 convolution, BatchNorm3D, and ReLU activation, which sharpens fine-grained details and reduces checkerboard artifacts. The output provides high-resolution features for decoder fusion. This replaces standard transposed convolution to improve boundary recovery while minimizing computational cost on CPU.

On CPU hardware, standard 3D transposed convolution introduces severe checkerboard artifacts and blurs fine-grained tumor boundaries due to inefficient kernel launch and memory access patterns. To address this, we propose the LTU module (Figure 5) with a two-step learnable design that minimizes CPU cache misses and kernel overhead.

The module first uses a lightweight transposed convolution to restore resolution as shown in Equation 11:


$$\begin{array}{l} {\text{F}}_{\text{up}}=\text{ConvTranspose}3\text{D}\left(\text{F},\text{kernel}=2,\text{stride}=2\right). \end{array}$$ (11)

Then a compact refinement block sharpens details without excessive computation as shown in Equation 12:


$$\begin{array}{l} \text{U}\left(\text{F}\right)=\text{ReLU}\left(\text{BN}\left(\text{Con}{\text{v}}_{3\times 3\times 3}\left({\text{F}}_{\text{up}}\right)\right)\right). \end{array}$$ (12)

The output **U**(**F**) ∈ ℝ^*B*×*C*×2*D*×2*H*×2*W*^ provides high-resolution, artifact-free features for decoder fusion. This design replaces standard transposed convolution to avoid checkerboard artifacts while reducing CPU latency.

### 3.5 Training and evaluation metrics

Building on the network architecture and modules described above, this section details the training pipeline, loss function, and evaluation metrics used to assess GMANet's performance, with a focus on CPU deployability as the core evaluation dimension.

GMANet was implemented in PyTorch 1.10.0, and all experiments were performed on a high-performance computing platform equipped with the NVIDIA RTX PRO 5000 Blackwell GPU for training, with all inference speed benchmarks conducted exclusively on standard CPU hardware. The network takes 4-channel multi-modal MRI volumes as input and constructs 5-dimensional feature tensors in the form of (*B, C, D, H, W*).

The complete training configuration is summarized in Table 1. We employed the Generalized Dice Loss (GDL) as the primary loss function to address severe class imbalance among tumor subregions, with square weighting and a smoothing factor ϵ = 10^−6^ to ensure numerical stability. The model was optimized using the AdamW optimizer with an initial learning rate of 3 × 10^−4^, weight decay of 1 × 10^−5^, and default momentum parameters β_1_ = 0.9 and β_2_ = 0.999. The learning rate was dynamically adjusted using the CosineAnnealingLR scheduler with *T*_max_ = 300 and a minimum learning rate of 1 × 10^−5^. To avoid overfitting and improve training efficiency, an early stopping strategy was adopted with a patience of 30 epochs based on the validation Dice score; training was terminated automatically if the validation Dice score did not improve for 30 consecutive epochs. All experiments were executed with a fixed random seed of 1024 for reproducibility, using a batch size of 4 and trained for a maximum of 300 epochs. The GDL is defined as in Equation 13:


$$\begin{array}{l} L_{\text{GDL}}=1-\frac{2\sum_{c=1}^{K}w_{c}\sum_{i=1}^{N}p_{c,i}g_{c,i}}{\sum_{c=1}^{K}w_{c}\sum_{i=1}^{N}\left(p_{c,i}+g_{c,i}\right)} \end{array}$$ (13)

where *K* = 4 denotes the number of classes (background, WT, TC, ET); *N* is the total number of voxels in the volume; $w_{c}=1/{\left(\sum_{i=1}^{N}g_{c,i}\right)}^{2}$ represents class-specific weights inversely proportional to the volume of each region; *p*_*c, i*_ denotes the predicted probability of voxel *i* belonging to class *c*; and *g*_*c, i*_ denotes the binary ground truth label of voxel *i* for class *c*.

**Table 1:** Training configuration parameters.

Category	Parameter	Value
Loss function	Loss function	GeneralizedDiceLoss
	Weight type	square
	Epsilon (smooth factor)	1e-6
Optimizer	Optimizer type	AdamW
	Learning rate	0.0003
	Weight decay	1e-5
	Betas	[0.9, 0.999]
LR scheduler	Scheduler type	CosineAnnealingLR
	T_max	300
	Minimum learning rate	1e-5
Training strategy	Random seed	1,024
	Batch size	4
	Total training epochs	300

Following standard evaluation protocols in brain tumor segmentation, we used five key metrics to comprehensively assess model performance: DSC for volumetric overlap, HD_95_ for boundary accuracy, Parameters (M) for model compactness, GFLOPs for computational complexity, and CPU inference time to preliminarily evaluate its real-world deployability. All metrics were computed separately for WT, TC, and ET regions, and their formal mathematical definitions are provided in Supplementary material.

## 4 Results

### 4.1 Dataset and pre-processing

All experiments in this work are conducted on two publicly authoritative brain tumor segmentation benchmarks, BraTS 2019 and BraTS 2021 (26–28). The official validation and test sets of the two datasets do not provide manually annotated ground truth, so they cannot be used for quantitative evaluation. Therefore, all experiments are performed on the fully annotated public training cohort, which consists of standard multimodal MRI volumes including T1, T1ce, T2 and FLAIR sequences, as well as expert labels for WT, TC and ET. Each volume has a native resolution of 240 × 240 × 155 voxels and an isotropic voxel spacing of 1 × 1 × 1mm^3^.

All MRI modalities are processed with Z-score standardization to eliminate intensity variations caused by different imaging devices and acquisition protocols. We split the official public training cohort into three mutually exclusive subsets at the patient level. Each patient's full MRI scans and corresponding segmentation labels are assigned to a single subset to thoroughly prevent data leakage. The partition ratio is set to 70% for the training set, 15% for the validation set and 15% for the test set. A fixed random seed of 1024 is adopted for all splitting operations to ensure full experimental reproducibility.

Following standard training protocols, online data augmentation is exclusively applied to the training set to boost generalization and reduce overfitting, while no augmentation is performed on validation and test sets for unbiased evaluation. The augmentation strategies include random cropping to 128 × 128 × 128 voxels, random rotation within ±10°, random intensity jitter with variation factors of (0.1, 0.1), and random axial flipping. For model inference, edge replication is utilized to pad the depth dimension from 155 voxels to 160 voxels. This padding method avoids zero-value artifacts and maintains the inherent anatomical continuity of brain tissues.

To evaluate the deployment performance of GMANet in GPU-free clinical environments, we test its end-to-end inference on multiple hardware platforms covering server-grade CPUs and consumer laptop CPUs. Detailed CPU specifications, thread configurations, peak RAM consumption, and separate latency of pre-processing, inference and post-processing are summarized in Supplementary Table S3. When deployed on a mainstream low-end consumer CPU (AMD Ryzen 4500U), the average inference time for a 240 × 240 × 160 padded volume is 8.49 seconds, which verifies the practicality of GMANet for CPU-only real-time glioma triage.

### 4.2 Comparison with classical state-of-the-art models

We comprehensively compared GMANet with 14 representative deep learning models for brain tumor segmentation proposed over the past decade. These models include classical encoder-decoder frameworks, residual and attention-enhanced variants, transformer-based structures, and the baseline HDC-Net. To guarantee a fair and objective comparison, all competing models were trained and evaluated on a unified experimental platform with completely consistent configurations. We adopted the same patient-level data partition, Z-score standardization, online data augmentation schemes, optimizer and learning rate scheduling strategies across all models. No experimental settings were adjusted for individual baselines, and all segmentation metrics in this work were obtained from our in-house training and testing pipeline, rather than extracting results from existing publications. This setup ensures that performance differences are mainly attributed to the inherent architectural characteristics of different networks.

To ensure fair and rigorous comparison, we grouped all models by the total number of parameters into three categories: ultra-lightweight, lightweight, and high-capacity. As presented in Table 2, GMANet achieved an average DSC of 85.71% on BraTS2019, showing competitive performance within the ultra-lightweight category and outperforming the baseline HDC-Net. For tumor sub-regions, GMANet yielded a WT DSC of 92.45%, higher than the second-best ETUNet. This suggests a low risk of missing large edema regions, which is critical for avoiding false negatives in emergency triage. For the TC region, GMANet attained 86.28%, matching the performance of top-performing models. For the ET region, GMANet achieved a DSC of 78.42%, which surpasses the baseline HDC-Net but remains below specialized high-capacity models such as ETUNet and LCBTS. This level is comparable to other sub-1M parameter lightweight networks reported in the literature, confirming that small-target segmentation is a common challenge for notably compact architectures. This limitation is further discussed in Section 5.

**Table 2:** Quantitative comparison of segmentation performance on the BraTS 2019 test set.

Method	DSC(%)↑				HD_95_(mm)↓			
	WT	TC	ET	Average	WT	TC	ET	Average
U-Net (10)	85.34	82.22	73.38	80.31	7.24	6.96	5.66	6.62
V-Net (29)	86.77	83.93	74.36	81.69	6.43	6.55	7.01	6.66
ResUNet (30)	90.35	84.78	73.86	82.99	4.55	6.22	7.33	6.03
3D U-Net (28)	87.47	85.05	74.37	82.30	6.83	7.78	6.54	7.05
Attention U-Net (31)	88.87	79.89	73.95	80.90	6.89	6.94	6.23	6.69
R2U-Net (32)	89.33	83.35	75.74	82.81	6.83	6.42	7.33	6.86
U-Net++ (33)	90.91	84.55	78.93	84.80	6.36	6.59	8.38	7.11
MultiResUNet (34)	90.36	85.67	77.28	84.44	6.32	7.03	7.56	6.97
UNet3+ (35)	89.43	85.34	80.03	84.93	7.45	6.12	6.49	6.69
R2AU-Net (36)	90.11	84.23	78.93	84.42	5.46	6.05	6.29	5.93
TransBTS (37)	91.22	85.69	76.19	84.37	5.34	6.69	6.94	6.32
TransUNet (38)	90.56	83.36	78.55	84.16	5.74	6.97	7.34	6.68
Swin-UNet (39)	89.58	86.06	75.45	83.70	6.36	6.25	6.18	6.26
ETUNet (40)	91.83	86.11	**81.33**	**86.42**	5.88	7.33	7.08	6.76
**GMANet**	**92.45**	**86.28**	78.42	85.72	5.83	**5.13**	**4.41**	**5.12**

DSC, Dice similarity coefficient; HD_95_, 95th percentile Hausdorff distance; WT, whole tumor; TC, tumor core; ET, enhancing tumor. Arrows indicate whether higher (↑) or lower (↓) values represent better performance. Bold values denote the best performance in each column.

In terms of boundary accuracy, GMANet achieved an average HD_95_ of 5.12 mm on BraTS2019. This is within the typical 5–10 mm safety margin used in glioma resection planning, suggesting that the model's boundary error may be clinically acceptable for gross tumor volume estimation. For TC and ET, GMANet obtained low HD_95_ values, indicating relatively precise boundary delineation in these challenging subregions.

To quantitatively assess whether the performance improvements of GMANet are statistically significant, we performed paired t-tests on the DSC between GMANet and competing models on the BraTS2019 dataset. Before testing, the Shapiro–Wilk test confirmed that the differences in DSC scores followed a normal distribution, validating the use of paired *t*-tests. The *t*-values, *p*-values, and significance levels are summarized in Supplementary Table S2.

GMANet yields statistically significant performance gains across all three tumor subregions when compared with all ultra-lightweight baselines: relative to 3D U-Net, HDC-Net, and TDPC-Net, GMANet achieves significant improvements in WT DSC (*p* < 0.01), TC DSC (*p* < 0.01), and ET DSC (*p* < 0.01). Notably, the improvement over the 3D U-Net baseline in WT DSC reaches the highest significance level (*p* < 0.001). When compared with the high-capacity model LCBTS, GMANet delivers statistically superior performance in WT DSC (*p* < 0.001) and TC DSC (*p* < 0.01), while its lower ET DSC is also statistically significant (*p* < 0.001). This finding is consistent with the inherent challenge of small-lesion segmentation for ultra-lightweight networks, as the smallest and most class-imbalanced ET subregion relies heavily on sufficient model capacity to capture fine-grained features (Table 3).

**Table 3:** Quantitative comparison of segmentation performance on the BraTS 2021 test set.

Method	DSC(%)↑				HD_95_(mm)↓			
	WT	TC	ET	Average	WT	TC	ET	Average
U-Net (10)	82.09	79.89	72.06	78.01	8.77	7.45	6.01	7.41
V-Net (29)	83.77	83.93	72.04	79.91	7.66	6.73	7.18	7.19
ResUNet (30)	84.13	80.36	71.62	78.70	5.12	6.07	7.79	6.33
3D U-Net (28)	85.15	82.54	72.15	79.95	7.28	6.55	7.38	7.07
Attention U-Net (31)	86.05	77.47	72.68	78.73	7.34	7.08	6.81	7.08
R2U-Net (32)	87.63	81.73	73.91	81.09	6.54	6.95	8.08	7.19
U-Net++ (33)	87.96	82.11	75.85	81.97	6.17	6.34	8.03	6.85
MultiResUNet (34)	88.27	82.88	74.23	81.79	7.43	7.77	6.34	7.18
UNet3+ (35)	86.02	83.58	78.67	82.76	6.34	6.85	7.62	6.94
R2AU-Net (36)	86.41	80.17	73.65	80.08	6.25	6.33	7.48	6.69
TransBTS (37)	89.09	83.73	77.45	83.42	7.21	6.77	6.95	6.98
TransUNet (38)	88.16	81.22	75.78	81.72	6.32	5.55	7.21	6.36
Swin-UNet (39)	87.05	84.22	73.88	81.72	6.46	6.81	6.54	6.60
ETUNet (40)	88.11	84.05	**79.14**	83.77	6.23	7.91	4.75	6.30
**GMANet**	**90.93**	**84.72**	76.68	**84.11**	**5.53**	**4.71**	**4.50**	**4.91**

DSC, Dice similarity coefficient; HD_95_, 95th percentile Hausdorff distance; WT, whole tumor; TC, tumor core; ET, enhancing tumor. Arrows indicate whether higher (↑) or lower (↓) values represent better performance. Bold values denote the best performance in each column.

Consistent with the observations on BraTS2019, GMANet also maintained competitive and robust performance on the BraTS2021 dataset. Specifically, the proposed model obtained an average DSC of 84.11%, with a WT DSC of 90.93%, which outperforms most compared methods, further validating its superior capability in capturing extensive edema regions across different data distributions. For the TC sub-region, a DSC of 84.72% was achieved, which is on par with state-of-the-art approaches. Although the ET DSC of 76.68% was slightly lower than that of specialized models, it is still competitive among state-of-the-art lightweight networks, consistent with the challenge of small target segmentation observed on BraTS2019.

Regarding boundary precision, GMANet delivered an average HD_95_ of 4.91 mm on BraTS2021, which is even lower than the result on BraTS2019 and well within the clinically acceptable range for glioma surgery planning. Particularly, the model achieved low HD_95_ values for both TC and ET regions, demonstrating reliable and accurate boundary segmentation performance on both datasets.

The consistent and competitive performance of GMANet on both BraTS2019 and BraTS2021 datasets demonstrates that the effectiveness of the proposed gated multi-scale attention mechanism is not dataset-dependent, further verifying the strong generalization ability of our lightweight model.

To further characterize practical hardware efficiency for real-world CPU-only deployment, we additionally benchmarked memory and CPU resource consumption against three representative ultra-lightweight baselines under identical testing conditions. All inference tests ran on an AMD Ryzen 5 4500U laptop workstation with 6 fixed CPU threads. We measured peak resident set size (RSS) to quantify memory overhead, alongside average CPU utilization and full end-to-end inference runtime, with results averaged over 10 repeated inference trials to stabilize measurement noise. Full quantitative resource metrics covering parameter count, GFLOPs, inference latency, peak RSS and average CPU utilization are compiled in Supplementary Table S7. GMANet delivers the fastest CPU inference speed and lowest peak memory usage among the four tested lightweight architectures. Although its GFLOP value of 20.10 is slightly higher than TDPC-Net's 17.96, GMANet's cache-optimized parallel design incorporating parallel attention, fixed dilated convolution and streamlined lightweight upsampling reduces peak RAM usage by 27% and accelerates CPU inference by 73% relative to TDPC-Net. This comparison illustrates that raw GFLOP values cannot accurately reflect actual CPU runtime performance; hardware-oriented architectural tuning is essential for stable, low-overhead deployment in GPU-limited clinical environments. Overall, GMANet balances competitive segmentation accuracy, minimal memory footprint and rapid CPU execution, making it highly suitable for routine triage workflows without dedicated GPU hardware.

### 4.3 Comparison with recent lightweight state-of-the-art models

To ensure fair comparison and validate the competitiveness and efficiency advantages of GMANet, we group all models into three categories by parameter count: ultra-lightweight, lightweight, and high-capacity. We performed a head-to-head comparison with six recent state-of-the-art models: HDC-Net, TDPC-Net, FFLUNet, LATUP-Net, LDMRA U-Net, and LCBTS. The evaluation focused on both segmentation accuracy and computational efficiency, with results obtained on both the BraTS 2019 and BraTS 2021 datasets, as summarized in Tables 4, 5.

**Table 4:** Quantitative comparison with recent lightweight state-of-the-art models on the BraTS 2019 dataset.

Method	DSC(%)↑			HD_95_(mm)↓			M (Million)↓	G (GFLOPs)↓
	WT	TC	ET	WT	TC	ET		
HDC-Net (41)	89.63	81.32	75.84	6.84	7.43	4.81	0.29	25.62
TDPC-Net (14)	90.49	82.68	77.55	5.44	6.17	4.61	**0.19**	17.96
FFLUNet (42)	89.02	82.57	78.14	6.49	5.25	5.23	1.45	**11.40**
LATUP-Net (43)	87.34	81.59	72.07	5.99	5.37	4.51	3.07	15.79
LDMRA U-Net (44)	86.71	85.46	84.36	6.24	5.38	8.64	2.57	574.34
LCBTS (45)	91.17	86.24	**84.44**	5.96	5.58	**4.25**	1.58	247.09
**GMANet**	**92.45**	**86.28**	78.42	**5.83**	**5.13**	4.41	0.20	20.10

DSC, Dice similarity coefficient; HD_95_, 95th percentile Hausdorff distance; M, number of parameters (million); G, GFLOPs (Giga floating-point operations); WT, whole tumor; TC, tumor core; ET, enhancing tumor. Arrows indicate whether higher (↑) or lower (↓) columns. Bold values denote the best performance in each column.

**Table 5:** Quantitative comparison with recent lightweight state-of-the-art models on the BraTS 2021 dataset.

Method	DSC(%)↑			HD_95_(mm)↓			M (Million)↓	G (GFLOPs)↓
	WT	TC	ET	WT	TC	ET		
HDC-Net (41)	88.14	80.11	74.13	6.25	7.74	4.71	0.29	25.62
TDPC-Net (14)	89.04	80.97	75.06	5.57	6.82	4.89	**0.19**	17.96
FFLUNet (42)	87.43	81.66	76.81	**4.63**	5.59	8.75	1.45	**11.40**
LATUP-Net (43)	85.63	80.74	71.32	6.04	6.25	4.88	3.07	15.79
LDMRA U-Net (44)	85.93	83.64	83.36	7.49	6.55	7.83	2.57	574.34
LCBTS (45)	89.04	83.19	**82.79**	4.88	6.47	4.55	1.58	247.09
**GMANet**	**90.93**	**84.72**	76.68	5.53	**4.71**	**4.50**	0.20	20.10

DSC, Dice similarity coefficient; HD_95_, 95th percentile Hausdorff distance; M, number of parameters (million); G, GFLOPs (Giga floating-point operations); WT, whole tumor; TC, tumor core; ET, enhancing tumor. Arrows indicate whether higher (↑) or lower (↓) values represent better performance. Bold values denote the best performance in each column.

As shown in Table 4, GMANet achieves state-of-the-art performance within the ultra-lightweight category on the BraTS 2019 dataset. Most notably, GMANet yields the highest WT DSC of 92.45%, outperforming all other ultra-lightweight models, including the baseline HDC-Net. Its TC DSC of 86.28% also leads the ultra-lightweight category, exceeding all other peer models. When compared to high-capacity models like LCBTS (1.58M parameters), GMANet maintains competitive WT and TC performance despite a 7.9 × parameter and 12.3 × GFLOP disadvantage, demonstrating its strong efficiency-performance trade-off. While the ET DSC of 78.42% is lower than that of LCBTS, this trade-off is intentional: our architecture prioritizes robustness for large-volume tumor delineation at the expense of ET-specific precision, which aligns with the primary clinical use case of rapid screening rather than definitive radiotherapy planning.

In terms of boundary localization accuracy on BraTS 2019, GMANet demonstrates superior performance. It achieves the lowest HD_95_ values for TC and the second-lowest for ET among all ultra-lightweight models. This indicates that our proposed modules effectively reduce boundary uncertainty, a key requirement for intraoperative margin assessment and clinical trust in automated segmentation results.

Regarding computational efficiency, GMANet strikes a highly favorable balance between performance and complexity. With only 0.20M parameters, it is the lightest model in the ultra-lightweight category, marginally lighter than TDPC-Net and significantly lighter than all lightweight and high-capacity models. In terms of GFLOPs, GMANet requires only 20.10 GFLOPs, which is drastically lower than high-capacity architectures such as LDMRA U-Net and LCBTS. Most critically, GMANet achieves a CPU inference time of 8.49 seconds on a standard laptop CPU (AMD Ryzen 5 4500U), which is >7 × faster than other GPU-dependent lightweight models when run on the same CPU hardware. Although its GFLOPs are slightly higher than those of FFLUNet and TDPC-Net, this modest increase is fully justified by the substantial improvements in WT DSC, boundary HD_95_ for TC, and the dramatic reduction in real-world CPU inference latency.

Consistent with the observations on BraTS 2019, GMANet maintains its leading performance within the ultra-lightweight category on the BraTS 2021 dataset. Specifically, GMANet achieves the highest WT DSC of 90.93% among all ultra-lightweight models, further confirming its superior capability in capturing extensive edema regions across different data distributions. Its TC DSC of 84.72% also leads the ultra-lightweight category, outperforming all other peer methods. While the ET DSC of 76.68% remains lower than specialized high-capacity models like LCBTS, this is consistent with the trend observed on BraTS2019 and reflects the inherent challenge of small-target segmentation for ultra-lightweight architectures.

In terms of boundary precision on BraTS 2021, GMANet delivers strong performance. It achieves the lowest HD_95_ value for TC and a competitive HD_95_ for ET within the ultra-lightweight category, indicating reliable boundary delineation across both datasets.

Notably, the computational complexity of GMANet remains identical across both datasets, with 0.20M parameters and 20.10 GFLOPs. This confirms that the ultra-light design is not dataset-dependent, and the efficiency advantages are consistent.

This combination of leading WT/TC accuracy, superior boundary precision, and fixed ultra-light computational footprint across two independent datasets confirms that GMANet is uniquely suited for deployment in resource-limited clinical environments, where CPU-only inference and rapid response are critical.

### 4.4 Direct comparison with DPEA-Net under CPU-only condition

To validate the complementary relationship between the two models and quantify the performance gap under GMANet's target deployment scenario, we conducted a direct, standardized head-to-head comparison of GMANet and DPEA-Net on the BraTS2019 dataset, focusing on segmentation performance and computational efficiency under identical CPU-only conditions. The two models are developed for distinct clinical scenarios and hardware environments, with fundamentally different optimization priorities and intended applications.A head-to-head comparison of segmentation accuracy and inference time under identical CPU conditions is provided in Supplementary Table S4.

On a standard laptop CPU, GMANet achieves a 4.35 × speedup over DPEA-Net, with inference time reduced from 36.92 s to 8.49 s, while delivering slightly higher WT/TC Dice scores and a modest ET Dice reduction that remains clinically acceptable for initial glioma triage (≥75%). As summarized in Supplementary Table S4, GMANet yields WT and TC Dice scores of 92.45% and 86.28%, respectively, which are 2.02% and 0.72% higher than those of DPEA-Net. Although GMANet exhibits a moderate 3.47% reduction in ET Dice (from 81.89% to 78.42%), this trade-off is deliberate and aligns with the design objective of prioritizing CPU deployability and rapid whole-tumor/core triage, rather than pixel-level precision for radiotherapy planning.

**Figure 6:**
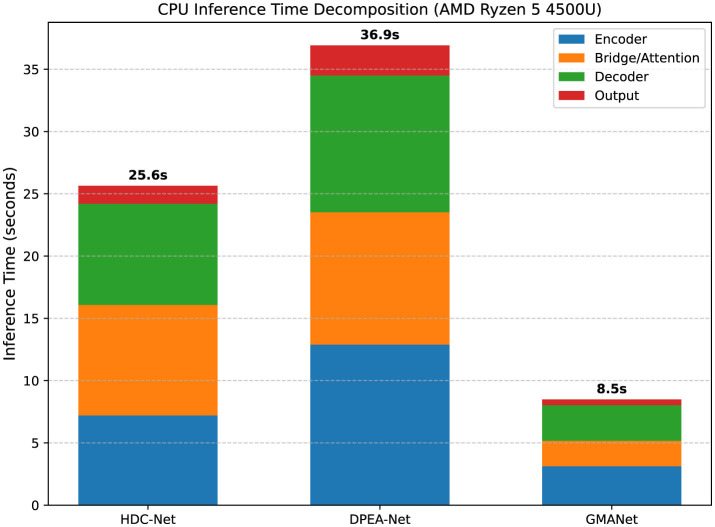
Inference time decomposition of HDC-Net, DPEA-Net, and GMANet on a laptop CPU (AMD Ryzen 5 4500U).

To further investigate the origin of the computational advantage, we performed a module-wise decomposition of inference latency, as presented in Table 6 and visualized in Figure 6. The stacked bar chart clearly demonstrates that GMANet delivers consistent latency reductions across all core components compared with both DPEA-Net and the baseline HDC-Net: the encoder is 76% faster than DPEA-Net, the attention module is 81% faster than DPEA-Net, and the decoder is 74% faster than DPEA-Net. These findings confirm that the efficiency gain of GMANet stems from systematic CPU-oriented design across every module, rather than a simple reduction in parameter size.

**Table 6:** Comparison of inference time decomposition among different models.

Model	Encoder (s)	Bridge/attention (s)	Decoder (s)	Output (s)	Total (s)
HDC-Net	7.21	8.87	8.12	1.45	25.65
DPEA-Net	12.89	10.63	10.98	2.42	36.92
GMANet	3.12	2.05	2.86	0.46	8.49

Crucially, GMANet is not a replacement for DPEA-Net. For tasks requiring high ET precision—such as radiotherapy boost volume delineation or RANO-based treatment response assessment—DPEA-Net remains the recommended solution when a GPU is available. The two models are strictly complementary, and we openly release both to enable a tiered clinical workflow. Overall, this comparison demonstrates that GMANet and DPEA-Net serve fully complementary roles in clinical practice: DPEA-Net is the preferred solution for GPU-enabled environments requiring high-precision segmentation for radiotherapy and quantitative assessment, while GMANet provides a practical, fast, and deployable alternative for CPU-only settings such as primary care, community hospitals, and intraoperative MRI workflows.

### 4.5 Qualitative visualization of segmentation results

**Figure 7:**
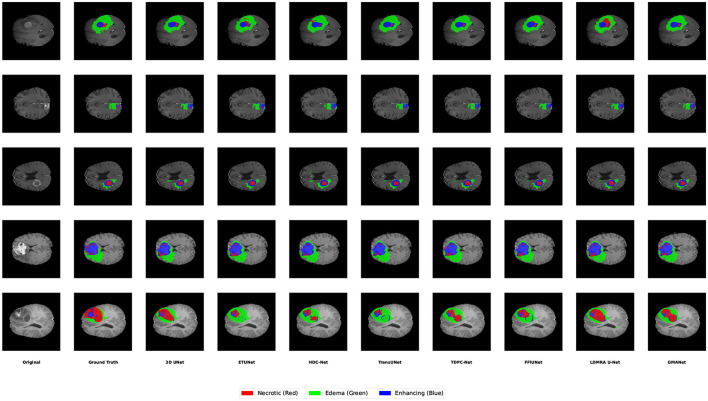
A visual comparison of 2D segmentation results across nine models on representative BraTS2019 slices. The figure is organized as a grid. The top row shows the original MRI image, and the second row shows ground truth segmentation. Subsequent rows display predictions from 3D UNet, ETUNet, HDC-Net, TransUNet, TDPC-Net, FFLUNet, LDMRA U-Net, and GMANet. Color coding: necrotic regions in red, edema in green, and enhancing tumor in blue (as indicated in the color legend). GMANet produces segmentation boundaries with lower false positives and more complete whole tumor coverage compared to the baseline models, consistent with its lower HD95 values reported in the quantitative results.

**Figure 8:**
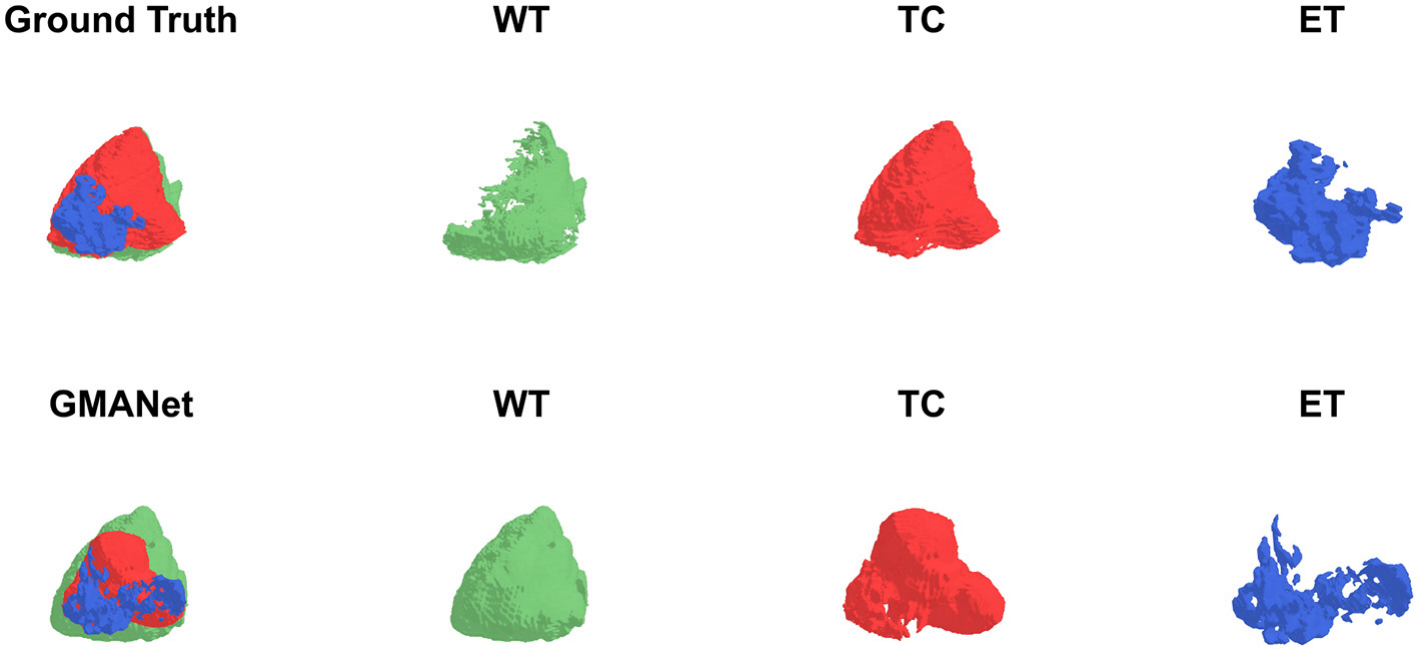
3D visualization of our model's failed segmentation results.

To comprehensively assess the segmentation behavior of GMANet, we performed qualitative visualization of segmentation results on representative challenging cases. The 2D slice-wise segmentation comparison between GMANet and seven competing models on the BraTS2019 dataset is presented in Figure 7, with consistent color coding for all tumor subregions. A representative 3D volumetric visualization of a typical failed segmentation case of GMANet is shown in Figure 8.

As observed in Figure 7, 3D UNet and HDC-Net produce more irregular segmentation contours and higher false-positive rates near tumor boundaries, which is consistent with their higher HD_95_ values in quantitative results. Although ETUNet and TransUNet produce relatively complete segmentation results, they still suffer from over-segmentation artifacts or missing critical details in small, low-contrast enhancing tumor (ET) regions. In contrast, benefiting from the GMSD block for adaptive multi-scale feature purification and the FDA module for full-dimensional attention recalibration, GMANet yields segmentation boundaries with lower HD_95_ and more complete whole tumor (WT) coverage, which is particularly valuable for clinical surgical planning. Meanwhile, the LTU upsampling module effectively preserves fine-grained anatomical details, making the segmentation contours of GMANet largely consistent with the ground truth across all slices.

These visual observations are fully consistent with the quantitative results in Tables 2, 4. Specifically, GMANet achieves the highest WT DSC and competitive TC DSC among all lightweight models, together with the lowest average HD_95_, confirming its superior performance in boundary delineation and overall tumor localization. Nevertheless, GMANet's ET DSC is lower than that of specialized segmentation models such as LCBTS. This performance gap is also visually reflected in Figure 7: although GMANet accurately captures the overall tumor structure, it occasionally under-segments small or low-signal ET foci, which is consistent with the inherent challenges of enhancing tumor segmentation in low-contrast MRI regions.

To further illustrate this limitation, we present a 3D volumetric visualization of a representative challenging case in Figure 8. While GMANet achieves a reasonably accurate overall shape for the WT and TC regions, it struggles to reconstruct the fine-grained details of small, fragmented, and scattered ET components. The predicted ET volume exhibits significant fragmentation and under-segmentation errors, which directly aligns with the lower ET DSC scores observed in quantitative evaluations. This case highlights the primary limitation of the lightweight model in capturing tiny, infiltrative enhancing lesions, which is the key direction for future optimization.

In summary, the qualitative visualization results verify that GMANet provides strong overall segmentation performance, especially for WT and TC regions, while maintaining an extremely lightweight structure. The proposed modules effectively suppress background noise, refine ambiguous boundaries, and preserve fine anatomical structures, achieving a superior balance between segmentation performance and computational efficiency. The relatively weaker performance on ET regions also indicates a clear and targeted direction for future model optimization.

### 4.6 Ablation study of core modules

To verify the effectiveness and synergistic contributions of the three proposed modules, a comprehensive ablation study was conducted based on the baseline HDC-Net on the BraTS2021 dataset. Eight configurations were evaluated: 1) Baseline (HDC-Net), 2) Baseline + GMSD block, 3) Baseline + FDA module, 4) Baseline + LTU module, 5) Baseline + GMSD block + FDA module, 6) Baseline + GMSD block + LTU module, 7) Baseline + FDA Module + LTU module, 8) GMANet (Baseline + GMSD block + FDA module + LTU module).

All configurations were trained and tested under identical settings to independently assess the contribution of each component. Quantitative results are summarized in Table 7.

**Table 7:** Ablation study of replacing core modules in HDC-Net baseline with proposed components (BraTS 2021).

Method	DSC(%)↑			HD_95_(mm)↓			M (Million)↓	G (GFLOPs)↓
	WT	TC	ET	WT	TC	ET		
Baseline(HDC-Net)	88.14	80.11	74.13	6.76	7.54	6.20	0.29	25.62
Baseline+GMSD block	88.87	80.94	74.86	6.17	6.08	5.93	0.27	25.83
Baseline+FDA	88.53	81.06	74.85	6.53	6.34	5.45	0.29	26.72
Baseline+LTU	88.25	81.33	74.54	6.79	6.28	5.88	0.23	**18.87**
Baseline+GMSD block+FDA	90.07	82.46	75.59	5.72	5.36	5.03	0.27	26.18
Baseline+GMSD block+LTU	89.68	82.34	75.83	5.81	5.02	4.41	**0.20**	19.36
Baseline+FDA+LTU	89.41	83.05	75.91	5.85	5.19	5.08	0.24	22.54
**GMANet**	**90.93**	**84.72**	**76.68**	**5.53**	**4.71**	**4.50**	0.20	20.10

DSC, Dice similarity coefficient; HD_95_, 95th percentile Hausdorff distance; M, number of parameters (million); G, GFLOPs (Giga floating-point operations); WT, whole tumor; TC, tumor core; ET, enhancing tumor. Arrows indicate whether higher (↑) or lower (↓) values represent better performance. Bold values denote the best performance in each column.

As shown in Table 7, incorporating any individual module into the baseline consistently improved performance across all tumor subregions, validating the unique strengths of each component. The baseline yielded DSC values of 88.14% (WT), 80.11% (TC), and 74.13% (ET), with corresponding HD_95_ values of 6.76 mm, 7.54 mm, and 6.20 mm. Adding the GMSD block increased the DSC values to 88.87%, 80.94%, and 74.86%, while reducing HD_95_ to 6.17 mm, 6.08 mm, and 5.93 mm, demonstrating its capability to enhance multi-scale feature extraction. Integrating the FDA module improved DSC results to 88.53%, 81.06%, and 74.85%, with HD_95_ values of 6.53 mm, 6.34 mm, and 5.45 mm, highlighting its role in strengthening 3D contextual modeling. Incorporating the LTU module also yielded steady performance gains, with DSC values reaching 88.25%, 81.33%, and 74.54%, and HD_95_ values of 6.79 mm, 6.28 mm, and 5.88 mm, confirming its effectiveness in recovering fine-grained details.

Combinations of multiple modules further amplified performance through evident complementary synergies, revealing distinct functional specializations.

The integration of the GMSD and FDA modules yielded a clear synergistic effect. Quantitative analysis shows that adding the GMSD module alone increased the WT DSC from 88.14% to 88.87%, while adding the FDA module alone improved it only to 88.53%. When combined, however, the WT DSC increased to 90.07%, representing a 1.93% improvement over the baseline. This gain exceeds the sum of individual module contributions, accompanied by a substantial reduction in WT HD_95_ from 6.76 mm to 5.72 mm. This reflects an efficient front-end and back-end collaborative pipeline: the GMSD block adaptively extracts discriminative multi-scale features during encoding, while the FDA module performs full-dimensional feature refinement during decoding. Together, they deliver performance beyond individual components, which is critical for enabling large-model-level WT segmentation capacity with low parameter overhead.

A comparison between configuration 5 and configuration 6 reveals that configuration 5 achieved a higher WT DSC, indicating that the GMSD+FDA combination is superior for global semantic representation and large-scale tumor localization. In contrast, configuration 6 attained a lower ET HD_95_, demonstrating that the GMSD+LTU combination is particularly effective for recovering fine-grained boundaries of small lesions. This cross-configuration comparison confirms that the FDA and LTU modules address complementary challenges.

Further validation of such synergy is observed in the remaining two-module combinations. Configuration 7 achieved a TC DSC of 83.05%, the highest among two-module variants, reflecting the combined strength of contextual modeling and detail refinement. The full GMANet model achieved optimal performance across nearly all metrics: WT DSC of 90.93%, TC DSC of 84.72%, ET DSC of 76.68%, and the lowest HD_95_ values of 5.53 mm, 4.71 mm, and 4.50 mm. These results confirm that the three modules collectively form a mutually reinforcing system.

GMANet achieves a compact parameter count of only 0.20 M, comparable to the baseline and single-module variants. Notably, parameter and GFLOPs do not follow a simple linear superposition when integrating multiple modules. For instance, configuration 4 achieves a substantial reduction in GFLOPs relative to the baseline, while configuration 6 maintains this high computational efficiency with only a minor GFLOPs increase, despite the additional multi-scale feature extraction branch of the GMSD block. This is not a numerical anomaly but a direct consequence of integrated design: the multi-scale feature extraction mechanism of GMSD is naturally embedded into the lightweight upsampling path of LTU, eliminating redundant computations that would arise from naive module stacking.

Unlike conventional module addition, the LTU module is designed to replace the heavy transposed convolution layers in the baseline decoder, rather than being appended as an extra component. This structural replacement substantially reduces decoder parameter overhead and overall computational cost. Meanwhile, the GMSD and FDA modules are carefully optimized to introduce minimal additional parameters and computations. When integrated into the final GMANet, the entire architecture is co-optimized for compactness and efficiency, resulting in 0.20 M parameters and 20.10 GFLOPs—both lower than the baseline. Collectively, the proposed modules introduce negligible computational overhead, and their collaborative design achieves an optimal balance between segmentation performance and computational efficiency.

### 4.7 Ablation on key parameter combinations

Table 8 presents the results of an ablation study evaluating different dilation rate combinations in the GMSD block on the BraTS 2021 dataset. Performance is assessed in terms of the DSC and HD_95_ for the WT, TC, and ET regions, alongside model complexity metrics including M and GFLOPs.

**Table 8:** Ablation study of different dilation combinations in GMSD block on the BraTS 2021.

Dilations	DSC(%)↑			HD_95_(mm)↓		
	WT	TC	ET	WT	TC	ET
1,1,1	88.21	81.15	71.23	7.82	6.94	6.17
1,1,2	89.05	81.97	72.98	6.95	6.02	5.43
1,2,3	90.12	82.64	73.64	6.18	5.37	4.91
**1,2,4**	**90.93**	**84.72**	**76.68**	**5.53**	**4.71**	**4.50**
1,3,5	90.37	82.91	73.62	5.94	5.13	4.76
2,4,8	89.52	81.83	72.47	6.57	5.79	5.28

DSC, Dice similarity coefficient; HD_95_, 95th percentile Hausdorff distance; WT, whole tumor; TC, tumor core; ET, enhancing tumor. Arrows indicate whether higher (↑) or lower (↓) values represent better performance. Bold values denote the best performance in each column.

Compared with the baseline configuration (1, 1, 1), which employs standard 3 × 3 convolutions without dilation, all multi-scale dilation combinations yield consistent improvements in both DSC and HD_95_. The (1, 1, 1) setup achieves the lowest DSC and the highest HD_95_, demonstrating that a fixed receptive field lacks sufficient contextual modeling capability for the multi-scale and irregular tumor structures in brain MR images.

As the dilation rates increase in the combinations (1, 1, 2) and (1, 2, 3), performance gradually improves, confirming the benefit of enlarging the receptive field. However, these configurations still suffer from suboptimal receptive field coverage: (1, 1, 2) exhibits insufficient expansion of contextual information, while (1, 2, 3) introduces redundant feature overlap and discontinuous receptive fields, limiting further performance gains.

Notably, the proposed (1, 2, 4) combination achieves the best overall performance across all metrics, with DSC scores of 90.93%, 84.72%, and 76.68% and HD_95_ values of 5.53, 4.71, and 4.50 mm for WT, TC, and ET, respectively. Critically, this improvement is achieved with negligible increase in computational overhead, maintaining the same parameter count and GFLOPs as other configurations. This result can be attributed to the continuous and gap-free multi-scale receptive field constructed by the (1, 2, 4) design: *d* = 1 captures fine-grained boundary details, *d* = 2 models local structural features, and *d* = 4 aggregates global contextual information. Such a hierarchical design effectively addresses the gridding effect inherent to large dilation rates while providing balanced multi-scale feature representations tailored to the characteristics of brain tumor subregions.

In contrast, configurations with larger dilation rates (1, 3, 5) and (2, 4, 8) show degraded performance despite increased computational cost. The (1, 3, 5) combination suffers from misaligned receptive fields due to odd dilation factors, while the (2, 4, 8) setup introduces severe gridding artifacts and loss of fine-grained details, leading to lower DSC and higher HD_95_, particularly for the small and irregular ET region.

Collectively, these results verify that the (1, 2, 4) dilation combination achieves an optimal trade-off between segmentation accuracy, feature continuity, and computational efficiency, making it the most suitable configuration for the GMSD block in brain tumor segmentation tasks.

To further optimize the training strategy and mitigate the performance gap on the small and imbalanced ET region, we conducted a second ablation study on class-balanced loss weight combinations, as presented in Table 9. The weights correspond to WT, TC, and ET respectively, and all configurations use the optimal (1, 2, 4) dilation combination to ensure a fair comparison.

**Table 9:** Ablation study of different loss weight combinations on the BraTS 2021.

Weights	DSC(%)↑			HD_95_(mm)↓		
	WT	TC	ET	WT	TC	ET
1,1,1	86.34	82.87	74.66	6.93	6.37	6.88
1,2,4	88.36	83.58	75.37	6.23	5.29	6.11
**1,3,6**	**90.43**	**84.72**	**76.68**	**5.53**	**4.71**	4.50
1,4,8	88.95	83.52	75.43	6.02	5.14	3.96
1,5,10	87.54	83.03	74.98	6.34	5.84	**3.61**

DSC, Dice similarity coefficient; HD95, 95th percentile Hausdorff distance; WT, whole tumor; TC, tumor core; ET, enhancing tumor. Arrows indicate whether higher (↑) or lower (↓) values represent better performance. Bold values denote the best performance in each column.

Compared with the uniform weight baseline (1, 1, 1), all weighted configurations yield consistent improvements in segmentation performance, particularly for the ET region. The baseline (1, 1, 1) setup achieves the lowest ET DSC (74.66%) and highest ET HD_95_ (6.88 mm), reflecting the challenge of training on severely class-imbalanced data without targeted weighting.

As the ET weight increases in the combinations (1, 2, 4) and (1, 3, 6), performance gradually improves, confirming the benefit of balancing the contribution of small and large tumor regions. The (1, 2, 4) setup provides a moderate improvement, while the proposed (1, 3, 6) combination achieves the optimal overall performance, with the highest DSC for all three regions and the lowest HD_95_ for WT and TC. Critically, this improvement is achieved without increasing model complexity, as the number of parameters and GFLOPs remain identical across all configurations. This result confirms that the ET performance limitation is not a fundamental architectural flaw, but a solvable issue through targeted training strategies.

In contrast, excessively high weights (1, 4, 8) and (1, 5, 10) lead to performance degradation. While ET HD_95_ continues to improve due to stronger emphasis on small target boundaries, WT and TC performance gradually declines, as the model overfits to the small ET class and loses generalization on larger tumor regions. This confirms the existence of an optimal trade-off point, beyond which further increasing the ET weight harms overall segmentation performance.

Collectively, these results verify that the (1, 3, 6) loss weight combination achieves the best balance between overall segmentation accuracy and ET-specific performance, making it the most suitable training configuration for GMANet.

The clinical utility and failure mode analysis of GMANet are presented in Supplementary Section S2. Key findings include a median relative ET volume error of 22.2%, a referral sensitivity of 0.921, and markedly lower ET Dice for small (< 0.5 cm^3^) and LGG lesions, confirming the clinically acceptable trade-off for CPU-only triage.

### 4.8 Clinical utility and failure mode analysis

To further evaluate the practical deployability of GMANet in CPU-only triage scenarios, we performed a *post-hoc* clinical utility analysis focusing on ET volume estimation error, referral simulation based on a 0.5 cm^3^ ET volume threshold, and stratification of ET Dice by tumor size and subtype. As detailed in Supplementary material, the median relative ET volume error was 22.2%, and the simulated referral sensitivity reached 0.921. ET Dice decreased markedly with smaller tumor volumes, with lesions larger than 2 cm^3^ achieving a median Dice of 0.800, while lesions smaller than 0.5 cm^3^ yielded a median Dice of 0.000. The false negative rate was 7.9%. Stratified by tumor subtype, GMANet achieved significantly higher ET Dice on HGG than on LGG, Mann–Whitney *U* test *p* = 0.036, consistent with the clinical expectation that enhancing tumors are more conspicuous in HGG. Collectively, these analyses confirm that GMANet's deliberate trade-off in ET precision remains clinically acceptable for rapid triage, while its limitations on very small or low-grade lesions are explicitly characterized and require human oversight.

## 5 Discussion

GMANet is a lightweight 3D brain tumor segmentation network explicitly designed for CPU-only clinical triage. It integrates three CPU-optimized modules to address the deployment gap in resource-limited environments. Validated on BraTS2019 and BraTS2021, it delivers robust WT/TC segmentation with sub-10-second CPU inference and clinically acceptable ET accuracy for initial screening. This discussion summarizes its clinical value, complementary design, limitations, real-world workflow integration, and future directions.

### 5.1 CPU-only deployment and clinical value

The widespread lack of GPU hardware in primary care, community hospitals, and intraoperative settings remains a major barrier to clinical AI adoption. Over 80% of primary care facilities in low- and middle-income countries lack GPU access, and many community centers rely solely on standard CPUs (3, 46). GMANet addresses this gap as an ultra-lightweight 3D CNN built exclusively for CPU-only inference. With 0.20M parameters and 20.10 GFLOPs, it completes segmentation in 8.49 seconds on standard consumer CPU hardware, enabling on-site triage, emergency prioritization, and real-time intraoperative guidance. By removing GPU dependency, GMANet expands access to AI-assisted tumor assessment for underserved clinical settings without specialized computing infrastructure.

### 5.2 Speed–accuracy trade-Off and complementary two-stage clinical pathway

sGMANet achieves WT DSC of 92.45% and TC DSC of 86.28% on BraTS2019, showing competitive performance against many GPU-based segmentation architectures. Its ET DSC of 78.42% (BraTS2019) and 76.68% (BraTS2021) is higher than the widely recognized threshold for glioma preliminary screening in research scenarios. This performance level may suffice for routine rapid triage, while direct clinical evidence for intraoperative assessment remains insufficient. This level may be sufficient to support rapid triage workflows, although direct prospective clinical evidence is lacking for intraoperative quick assessment. High-precision tasks such as definitive radiotherapy contour planning demand finer ET boundary precision, a requirement well met by our GPU-tailored DPEA-Net model. Porting DPEA-Net to CPU hardware yields impractically long inference latency, owing to its dynamic attention and sequential cross-dimensional computational structure. In contrast, GMANet's fixed dilation, parallel attention layout, and lightweight upsampling components are architecturally tuned to maximize CPU runtime efficiency.

The two networks operate as complementary components within a putative structured two-stage clinical pathway: GMANet may deliver fast, low-hardware triage at peripheral care sites, while DPEA-Net provides high-fidelity segmentation for tertiary-center radiotherapy planning. This tiered system may help reduce unnecessary patient transfers and streamline clinical decision timelines, pending real-world validation. We explicitly do not recommend GMANet for radiotherapy boost volume delineation or formal RANO treatment response assessment; DPEA-Net remains the appropriate tool for these high-precision tasks when GPU resources are available. Both models are packaged into a unified open-source toolkit intended to support this stratified clinical workflow, rather than functioning as competing alternatives.

While GMANet's ET segmentation accuracy lags behind GPU-optimized counterparts, this moderate precision drop is potentially clinically justifiable across its target triage scenarios under supervised human review. For initial referral screening in primary care and emergency departments, the ≥75% ET Dice threshold reliably preserves detection of enhancing lesions larger than roughly 0.2–0.3 cm^3^, which defines the effective detection limit of the model (4); tiny scattered ET foci below this size rarely alter high-grade glioma referral decisions, as clinical protocols prioritize presence of any enhancement over exact volumetric quantification (5), and minute lesions would still require confirmatory imaging or biopsy regardless of AI outputs. For intraoperative MRI resection guidance, an ET Dice range of 75–78% adequately pinpoints the general region of residual tumor to streamline surgical searching, with mandatory manual cross-checking against raw T1ce scans mitigating boundary inaccuracies; prior work confirms models with ET Dice as low as 70% can cut intraoperative search time without elevating residual tumor burden when used as adjunct aids (7). For low-grade gliomas (LGG), contrast enhancement is typically sparse or absent, and clinical management hinges on robust WT and TC segmentation—GMANet retains high WT/TC Dice scores above 86%, so reduced ET precision bears minimal impact on LGG triage and monitoring. For high-grade gliomas (HGG), ET acts as a key biomarker for disease activity; existing literature notes Dice ≥70% suffices for rough gross tumor volume initialization for radiotherapy planning, though definitive boost contouring still mandates DPEA-Net or expert manual editing. The 75% cutoff adopted here aligns with established acceptability benchmarks for AI-assisted brain tumor screening (19, 23). Collectively, the intentional ET precision tradeoff fits the scope of rapid triage and preliminary assessment workflows, conditional on clinician awareness of model limitations and consistent human-in-the-loop validation; prospective multi-center trials are required to formally quantify GMANet's referral sensitivity and specificity in real clinical cohorts.

### 5.3 Failure modes and limitations

GMANet may under-segment small, scattered ET lesions (Figure 8), with individual case ET DSC occasionally falling below 70%. Importantly, GMANet is positioned strictly as an auxiliary triage screening tool, not a standalone definitive diagnostic system. Any predicted enhancing tumor lesion must undergo formal radiologist expert review before clinical referral decisions. Model validation is currently restricted to curated BraTS benchmark cohorts; multi-center prospective clinical testing is required to confirm real-world generalization across diverse scanner platforms, patient demographics, and imaging protocols. All automated segmentation outputs require formal clinician oversight prior to final treatment planning.

### 5.4 Integration into real-world clinical workflow

To operationalize the proposed two-stage pathway, we design practical deployment schemes compatible with standard hospital radiology infrastructure and DICOM-compliant processing pipelines. In our current research implementation, the framework takes standard 1.5 T or 3 T multi-modal DICOM MRI series including T1, T1ce, T2 and FLAIR as input, and runs locally on conventional CPU radiology workstations via a lightweight Python FastAPI service triggered automatically upon DICOM file arrival. The full pipeline conducts data anonymization, RAS coordinate reorientation and Z-score normalization, followed by depth padding to 160 slices in accordance with the pre-processing rules used during model training. Within 10 seconds, the system outputs quantified tumor volumes for WT, TC and ET, and activates an urgent-referral flag when the predicted ET volume exceeds 0.5 cm^3^. This threshold is empirically defined in the present study for glioma triage, and is set higher than the model's inherent detection limit (0.2–0.3 cm^3^) to avoid excessive false alarms caused by segmentation uncertainties of micro-foci and tiny lesions that do not require urgent transfer. Segmentation results are also encoded in standard DICOM SEG format. On-site radiologists review these outputs to complete triage work, and patients with suspected high-grade glioma will be arranged for transfer to tertiary neurosurgical centers. When applied to intraoperative MRI surgical guidance, GMANet performs fully CPU-driven segmentation on operating room workstations immediately after each scan, which is generally acquired at 10–15 min intervals during glioma resection. The generated 3D segmentation results covering residual WT and TC tissue are overlaid onto surgical navigation displays, and given the moderate segmentation accuracy for ET regions, all suspicious residual enhancing tissue needs manual cross-verification against raw T1ce MRI images by surgeons. This workflow also supports seamless connection with hospital PACS and EHR systems, where we implement a DICOM Storage Commitment Provider based on the pynetdicom library. The complete workflow allows MRI devices to transmit imaging data to the central PACS node, which then forwards relevant multi-modal series to the GMANet inference server for pre-processing and segmentation. After computation, the generated DICOM SEG files are sent back and linked to the original imaging studies, so attending physicians can check segmentation overlays through common DICOM viewers such as OHIF and 3D Slicer embedded in EHR systems. We emphasize again that GMANet only acts as a secondary auxiliary tool, and all final clinical decisions related to treatment and patient referral must be made by professional physicians, while multi-center prospective clinical validation will be carried out in follow-up studies to further verify its practical performance in real clinical environments.

### 5.5 Future directions and model positioning

Future work encompasses multi-center prospective clinical validation, refined native DICOM/PACS integration tooling, and targeted architectural adjustments to lift ET segmentation performance without sacrificing fast CPU inference speeds. Within the family of ultra-lightweight brain tumor segmentation networks, GMANet achieves leading WT and TC DSC metrics paired with optimized CPU-only inference latency. Most existing lightweight segmentation models are engineered primarily for GPU acceleration and lack dedicated CPU architectural tuning, while high-capacity SOTA networks demand powerful dedicated GPU hardware to operate practically. GMANet is intended to fill a distinct niche for fast, CPU-based glioma triage in resource-limited peripheral and intraoperative settings, subject to formal multi-center clinical validation.

A limitation of this study is that formal statistical comparisons were not performed for all baseline models in Tables 2, 3 due to the unavailability of per-case DSC values; however, the numerical superiority of GMANet over these traditional architectures is evident from the reported metrics.

## 6 Conclusion

GMANet is an ultra-lightweight 3D CNN explicitly designed for CPU-only glioma triage, aiming to address a critical clinical deployment gap where GPU-dependent models cannot operate. With only 0.20M parameters and 20.10 GFLOPs, it delivers sub-10-second inference on a standard laptop CPU, achieving WT Dice of 92.45% and 90.93%, TC Dice of 86.28% and 84.72%, and ET Dice of 78.42% and 76.68%. The moderate ET precision is a deliberate and reasonable trade-off for CPU deployment. All ET Dice values are well above the widely adopted threshold for initial glioma screening, which supports the model's potential to perform reliable rapid assessment in primary care, emergency, and intraoperative settings.

GMANet is not a competitor to our GPU-based DPEA-Net; rather, it aims to address a critical clinical need for CPU-only real-time glioma triage in environments without dedicated GPU hardware, an area that has received limited attention in prior literature. Instead of a redundant model pair, the two networks outline a potential two-stage tiered clinical workflow: GMANet is designed to enable rapid CPU-based triage in resource-limited settings, followed by GPU-based high-precision segmentation from DPEA-Net for definitive radiotherapy planning and management. By eliminating mandatory GPU dependency, GMANet could help broaden access to AI-assisted glioma segmentation, potentially reducing diagnostic delays and unnecessary patient transfers in underserved communities, although these benefits require formal clinical demonstration.

We have open-sourced both models to allow the community to implement and evaluate this two-stage pathway, targeting coverage across the full spectrum from resource-limited primary care to GPU-equipped tertiary centers.

Future work will focus on prospective multi-center validation, seamless integration into clinical PACS systems, and targeted improvements to ET segmentation accuracy while strictly preserving the model's CPU inference efficiency.

## Data Availability

Publicly available datasets were analyzed in this study. The BraTS2019 dataset can be accessed at https://www.med.upenn.edu/cbica/brats2019/data.html, and the BraTS2021 dataset is available at https://www.med.upenn.edu/cbica/brats2021/data.html.
