# Supplementary material for: GMANet: a CPU-only ultra-lightweight 3D CNN for real-time glioma triage in resource-limited environments
Source: Front Neurol. 2026 Jul 23;17:1894796. doi: 10.3389/fneur.2026.1894796 (PMC13445960; doi:10.3389/fneur.2026.1894796)
Supplement: Supplementary file 1 [file Data_Sheet_1.pdf]

# Supplementary Material

## 1 DETAILED METHOD

The official code of GMANet: Gated Multi-Scale Attention Network is publicly available at https://github.com/jingxuerong/GMANet. This repository provides the PyTorch implementation for lightweight 3D brain tumor segmentation, supporting fast CPU inference and reproducible experiments on BraTS2019 and BraTS2021 datasets.

### 1.1 PGConv3D Module Principle and Mathematical Formulas

PGConv3D is a lightweight gated feature enhancement module tailored for 3D medical image segmentation. It integrates channel-split partial convolution, anisotropic spatial convolution, and adaptive gated residual fusion. Only 1/4 of the input channels are convolved to reduce computation, while the others preserve intrinsic information. An axial-separated convolution captures 3D spatial context, and a learnable gate adaptively fuses raw and transformed features for robust representation.

Let the input 3D feature be $\boldsymbol{X} \in \mathbb{R}^{B\times C\times D\times H\times W}$, where $B$, $C$, $D$, $H$, $W$ denote batch size, channels, depth, height, and width. Let $w_1, w_2$ be learnable scalar weights, $\sigma$ denote the Sigmoid function, $\odot$ denote element-wise multiplication, Conv denote 3D convolution, BN denote batch normalization, split$(\cdot)$ denote channel-wise split, and cat$(\cdot)$ denote channel-wise concatenation.

The formulation of Partial_conv3 is defined as:

$$
\begin{cases}
C_c = \lfloor C/4 \rfloor, \quad C_r = C - C_c \\
\boldsymbol{X}_1, \boldsymbol{X}_2 = \text{split}(\boldsymbol{X}, [C_c, C_r]) \\
\boldsymbol{X}_1' = w_1 \cdot \text{Conv}_{3\times3\times3}(\boldsymbol{X}_1),\ \boldsymbol{X}_2' = w_2 \cdot \boldsymbol{X}_2 \\
\boldsymbol{X}_f = \text{cat}(\boldsymbol{X}_1', \boldsymbol{X}_2') \\
\boldsymbol{X}_t = \text{Conv}_{1\times3\times3}\big(\sigma\big(\text{BN}\big(\text{Conv}_{3\times3\times1}(\boldsymbol{X}_f)\big)\big)\big) \\
\boldsymbol{G} = \sigma\big(\text{BN}\big(\text{Conv}_{1\times1\times1}\big(\text{cat}(\boldsymbol{X}, \boldsymbol{X}_t)\big)\big)\big) \\
\boldsymbol{X}_{\text{out}} = \boldsymbol{G} \odot \boldsymbol{X} + (1 - \boldsymbol{G}) \odot \boldsymbol{X}_t
\end{cases}
$$

1. **Channel Split**: Input features are partitioned into a partial convolutional branch and an untouched branch.
2. **Weighted Feature Transform**: The partial branch is convolved, and both branches are scaled by learnable weights.
3. **Anisotropic Convolution**: $3 \times 3 \times 1$ and $1 \times 3 \times 3$ convolutions extract spatial and depth-wise context.
4. **Gate Generation**: A spatial attention gate is generated by concatenating raw and transformed features.
5. **Gated Fusion**: The gate adaptively fuses original and enhanced features for the final output.

### 1.2 CDRSEA Module vs. FDA Module

To explicitly clarify the fundamental architectural differences between the attention module in our prior work (CDRSEA, DPEA-Net) and the proposed FDA module (GMANet), we provide a side-by-side comparative analysis in Table S1, as the two modules are designed for entirely distinct optimization

objectives: CDRSEA is built for high-precision boundary refinement on GPU hardware, while FDA is engineered for maximum CPU inference efficiency. As detailed in the table, the core distinctions are fourfold: first, in design principle, CDRSEA relies on cross-dimensional feature interaction to simulate multi-planar reconstruction (MPR) for precise boundary modeling, while FDA adopts parallel channel-spatial-depth attention to efficiently capture global tumor context; second, in execution mode, CDRSEA uses sequential grouped convolutions to preserve fine-grained boundary details, while FDA implements fully parallel branches to eliminate sequential kernel launch overhead on CPUs; third, in hardware efficiency, CDRSEA has lower CPU efficiency due to its sequential computation pattern, while FDA is optimized for higher CPU performance with parallel, cache-friendly operations; finally, in clinical target, CDRSEA is optimized for TC/ET boundary refinement for radiotherapy planning, while FDA prioritizes whole-tumor coverage and slice continuity for rapid triage screening. These differences confirm that the FDA module is not a minor modification of CDRSEA, but a complete redesign tailored to the CPU-only clinical triage use case of GMANet.

**Table S1.** Comparative analysis of CDRSEA (DPEA-Net) and FDA (GMANet).

| Aspect | CDRSEA (DPEA-Net) | FDA (GMANet) |
|---|---|---|
| Design principle | Cross-dimensional interaction (MPR simulation) | Parallel channel-spatial-depth attention |
| Execution mode | Sequential grouped convolutions | Fully parallel branches |
| CPU efficiency | Lower (sequential kernel launches) | Higher (parallel, cache-friendly) |
| Target subregion | TC/ET boundary refinement | Whole-tumor coverage + slice continuity |

## 1.3 Definition of Evaluation Metrics

This section provides the formal definitions, mathematical formulations, and clinical roles of the key evaluation metrics used in this study, including DSC, $\text{HD}_{95}$, M, and GFLOPs.

DSC is the primary evaluation metric for medical image segmentation, which quantifies the volumetric overlap between the automated segmentation result and the ground truth annotation. It ranges from 0 to 1, where a higher value indicates better segmentation accuracy. The mathematical formulation is defined as:

$$\text{DSC} = \frac{2 \times TP}{2 \times TP + FP + FN}, \tag{S1}$$

where $TP$, $FP$, and $FN$ denote true positives, false positives, and false negatives, respectively.

$\text{HD}_{95}$ measures the boundary alignment between the predicted segmentation and the ground truth, reflecting the accuracy of tumor boundary delineation. It is defined as the 95th percentile of the maximum Euclidean distance between two point sets, reducing the impact of outliers. A lower $\text{HD}_{95}$ value indicates sharper and more accurate boundary prediction. The formal definition is:

$$\text{HD}_{95}(S, G) = \max \left\{ \underset{s \in S}{\text{Percentile}} \min_{g \in G} \|s - g\|_2, \ \underset{g \in G}{\text{Percentile}} \min_{s \in S} \|s - g\|_2 \right\}, \tag{S2}$$

where $S$ denotes the predicted set and $G$ denotes the ground truth set.

The total number of trainable parameters (Params) measures the model scale and is calculated by summing the parameters of all convolutional, fully connected, and attention layers. For a standard 3D convolution

layer with kernel size $k_d \times k_h \times k_w$, input channels $C_{in}$, and output channels $C_{out}$, the number of parameters is:

$$\text{Params}_{\text{Conv3d}} = C_{in} \times C_{out} \times k_d \times k_h \times k_w + C_{out}, \tag{S3}$$

where the bias term is included. The total parameters of the network is the sum of all layers, reported in millions (M).

GFLOPs (Giga Floating-point Operations) quantify the computational cost during one forward pass. For a 3D convolutional layer, the floating-point operations are computed as:

$$\text{FLOPs}_{\text{Conv3d}} = 2 \times C_{in} \times C_{out} \times k_d \times k_h \times k_w \times D \times H \times W, \tag{S4}$$

where $D, H, W$ are the output spatial dimensions. The total computational cost is the sum of all layers, converted into giga FLOPs (1 GFLOP = $10^9$ FLOPs).

### 1.4 Training and Inference Pipeline of GMANet

To provide a clear and reproducible workflow of the entire study, we summarize the complete training, optimization, and validation pipeline of GMANet as a formal algorithm. This pipeline unifies data preprocessing, online augmentation, model training, loss calculation, parameter optimization, and model inference, consistent with all experimental settings described in the previous sections. The overall training and inference procedure is formally presented in Algorithm 1.

**Algorithm 1** Training and Validation Pipeline of GMANet

**Require:** $(X_{\text{tra}}, Y_{\text{tra}})$: training dataset,
$\theta$: trainable parameters of GMANet,
$X_{\text{val}}$: validation dataset
**Ensure:** $\hat{Y}_{\text{val}}$: predicted segmentation masks
**Step 1:** Data Preprocessing and Augmentation
**for** $i = 1, 2, \ldots, N_{\text{tra}}$ **do**
    Normalize each modality independently using Z-score standardization
    Apply random crop, rotation, intensity jitter, and flipping
**end for**
**Step 2:** Model Training and Optimization
**for** $epoch = 1, 2, \ldots, epoch_{\text{i}}$ **do**
    **for** each mini-batch $(X_b, Y_b)$ in training set **do**
        Predict probability maps $\hat{Y}_b \leftarrow \text{GMANet}(X_b \mid \theta)$
        Compute $\mathcal{L}_{\text{GDL}}$ using Generalized Dice Loss
        Update parameters $\theta \leftarrow \theta - \eta \cdot \nabla \mathcal{L}_{\text{GDL}}(\theta)$
        Perform gradient clipping and update learning rate scheduler
    **end for**
**end for**
**Step 3:** Save the best-performing model $\theta^*$
**Step 4:** Inference on Validation Data
**for** $j = 1, 2, \ldots, N_{\text{val}}$ **do**
    Pad volume depth from 155 to 160 for network compatibility
    Predict segmentation $\hat{Y}_j \leftarrow \text{GMANet}(X_j \mid \theta^*)$
**end for**
**return** $\hat{Y}_{\text{val}}$

## 1.5 Inference Speed and Deployment Feasibility of GMANet

To keep the main manuscript focused on the core scientific contributions, we have moved comprehensive inference performance results into the supplementary materials. First, we evaluated the overall end-to-end inference speed of GMANet on a variety of hardware, including multiple CPUs and a professional GPU. The overall runtime and corresponding standard deviation for each platform are summarized in Supplementary Table S2. As shown in the table, the average end-to-end inference time on the mainstream consumer laptop (AMD Ryzen 5 4500U) reaches 8.49 seconds, which fully verifies the practicality of GMANet for real-time clinical inference in GPU-limited environments.

To further analyze the pipeline latency and hardware resource consumption, we split the whole workflow into preprocessing, model inference and postprocessing stages, and recorded the number of running threads as well as peak RAM usage on each CPU platform. The detailed segmented runtime and resource overhead are presented in Supplementary Table S3. All tested CPU devices maintain low memory occupancy, and the model achieves stable acceleration as the CPU performance improves, further demonstrating the excellent cross-platform deployment capability of the proposed ultra-lightweight network.

To further compare hardware efficiency between GMANet and other representative lightweight glioma segmentation architectures, we benchmarked memory footprint, CPU utilization, computational complexity

and end-to-end inference latency under identical AMD Ryzen 5 4500U CPU conditions, with full results presented in Table S4.

**Table S2.** Inference time and standard deviation of GMANet on different hardware environments.

| Model | Hardware Environment | Input Size | Inference Time (s) | Standard Deviation |
|---|---|---|---|---|
| GMANet | Intel(R) Xeon(R) Gold 6548Y+ | $240 \times 240 \times 160$ | 1.48 | 0.37 |
| GMANet | NVIDIA RTX PRO 5000 Blackwell GPU | $240 \times 240 \times 160$ | 0.35 | 0.02 |
| GMANet | AMD EPYC 48-Core Processor | $240 \times 240 \times 160$ | 5.19 | 0.63 |
| GMANet | Intel Xeon Platinum 8260L CPU | $240 \times 240 \times 160$ | 6.72 | 0.81 |
| GMANet | AMD Ryzen 5 4500U with Radeon Graphics | $240 \times 240 \times 160$ | 8.49 | 1.23 |

**Table S3.** Segmented inference performance and resource consumption on different CPU hardware platforms

| Hardware Platform | CPU Configuration | Threads | Peak RAM (GB) | Preprocessing (s) | Inference (s) | Postprocessing (s) |
|---|---|---|---|---|---|---|
| Consumer Laptop | AMD Ryzen 5 4500U | 6 | 2.5 | 1.18 | 6.13 | 1.18 |
| Mid-range Server | Intel Xeon Platinum 8260L | 32 | 3.4 | 0.85 | 4.126 | 0.84 |
| High-end Server | Intel Xeon Gold 6548Y+ | 64 | 4.1 | 0.33 | 0.82 | 0.32 |

*Abbreviations:* All tests adopt the input size of $240 \times 240 \times 160$. Time values are presented as mean $\pm$ standard deviation. Peak RAM represents the maximum memory occupancy during the whole inference pipeline.

**Table S4.** Resource consumption comparison on AMD Ryzen 5 4500U CPU platform

| Model | Params (M) | GFLOPs | CPU Inference Time (s) | Peak RSS (GB) | Avg CPU Util (%) |
|---|---|---|---|---|---|
| HDC-Net | 0.29 | 25.62 | 25.65 | 2.34 | 72 |
| TDPC-Net | 0.19 | 17.96 | 31.28 | 2.56 | 68 |
| FFLUNet | 1.45 | 11.40 | 45.30 | 3.12 | 65 |
| GMANet | 0.20 | 20.10 | 8.49 | 1.87 | 78 |

*Abbreviations:* All tests adopt input volume size $240 \times 240 \times 160$, running with 6 fixed CPU threads. RSS = resident set size, representing peak occupied memory during full inference pipeline. Time values are averaged over 10 repeated independent inference runs to eliminate random system fluctuation.

## 1.6 Statistical Significance Analysis

To evaluate whether the performance differences between GMANet and competing models are statistically significant, we conducted paired t-tests on the DSC across the three subregions on the BraTS2019 dataset. The Shapiro-Wilk test confirmed that the distribution of differences between paired samples was normal, supporting the validity of the paired t-test. All t-values, p-values, and significance levels are detailed in Supplementary Table S5.Key observations from the statistical analysis are as follows:

WT and TC: GMANet outperformed all baseline methods with statistically significant improvements ($p < 0.01$). Notably, against 3D U-Net and LCBTS, the improvements were highly significant ($p < 0.001$). ET: GMANet achieved significantly higher DSC scores than most models, including 3D U-Net, HDC-Net,

and TDPC-Net ($p < 0.01$). However, it was significantly outperformed by ETUNet and LCBTS ($p < 0.001$), which are specifically designed to prioritize small-lesion segmentation. This trade-off is consistent with the design constraints of ultra-lightweight networks.

**Table S5.** Statistical comparison of GMANet against baseline methods on the BraTS 2019 dataset.

| Comparison | Metric | $t$-value | $p$-value | Significance | Cohen's $d$ | 95% CI |
|---|---|---|---|---|---|---|
| GMANet vs. 3D U-Net | WT_DSC | 9.8047 | $< 0.0001$ | *** | 1.1890 | $[0.0397, 0.0600]$ |
| | TC_DSC | 2.9680 | 0.0033 | ** | 0.3599 | $[0.0042, 0.0216]$ |
| | ET_DSC | 2.8290 | 0.0053 | ** | 0.3431 | $[0.0076, 0.0442]$ |
| GMANet vs. ETUNet | WT_DSC | 2.9174 | 0.0048 | ** | 0.3538 | $[0.1117, 0.5959]$ |
| | TC_DSC | 2.6718 | 0.0092 | ** | 0.3240 | $[0.0819, 0.5661]$ |
| | ET_DSC | -3.6977 | 0.0004 | *** | -0.4484 | $[-0.6905, -0.2063]$ |
| GMANet vs. HDC-Net | WT_DSC | 3.1507 | 0.0024 | ** | 0.3821 | $[0.0124, 0.0552]$ |
| | TC_DSC | 2.7347 | 0.0080 | ** | 0.3316 | $[0.0200, 0.1282]$ |
| | ET_DSC | 2.8880 | 0.0052 | ** | 0.3502 | $[0.0170, 0.0933]$ |
| GMANet vs. TDPC-Net | WT_DSC | 3.7808 | 0.0030 | ** | 0.4585 | $[0.0187, 0.0605]$ |
| | TC_DSC | 2.9380 | 0.0045 | ** | 0.3563 | $[0.0117, 0.0615]$ |
| | ET_DSC | 2.7450 | 0.0065 | ** | 0.3329 | $[0.0057, 0.0358]$ |
| GMANet vs. LCBTS | WT_DSC | 3.5402 | 0.0007 | *** | 0.4293 | $[0.0056, 0.0201]$ |
| | TC_DSC | 2.6670 | 0.0094 | ** | 0.3234 | $[0.0003, 0.0017]$ |
| | ET_DSC | -6.2339 | $< 0.0001$ | *** | -0.7560 | $[-0.1107, -0.0625]$ |

*Abbreviations:* vs., versus; WT, Whole Tumor; TC, Tumor Core; ET, Enhancing Tumor; DSC, Dice Similarity Coefficient.

*Significance levels:* * $p < 0.05$, ** $p < 0.01$, *** $p < 0.001$.

*Note:* Positive $t$-values indicate that GMANet outperforms the comparison method, and negative values indicate the opposite.

## 1.7 DSC Distribution Analysis via Box Plots

To further evaluate the performance stability and generalization capability of the proposed GMANet, we conducted a per-sample statistical analysis of DSC scores across all test cases on the BraTS2021 dataset. The distribution of DSC scores for GMANet and the baseline HDC-Net across the WT, TC, and ET subregions are visualized in Figure S1, with box plots depicting the median, interquartile range (IQR), and outlier samples for each metric.

As shown in Figure S1, GMANet achieves consistent performance improvements over the baseline HDC-Net across all three tumor subregions. For the WT region, GMANet exhibits a higher median DSC score with a narrower interquartile range, indicating more stable and consistent whole-tumor segmentation performance across diverse test cases. For the TC region, GMANet not only increases the median DSC score but also significantly reduces the number of low-score outlier samples, which demonstrates its enhanced robustness in handling cases with highly variable tumor core morphology. For the ET region, GMANet yields a higher mean DSC score and a more concentrated distribution compared to HDC-Net, with fewer extreme low-score outliers, confirming its improved capability to capture small and fragmented enhancing lesions.

Notably, GMANet outperforms HDC-Net in both the central tendency and the distribution compactness for all three subregions. The narrower interquartile range indicates that GMANet maintains high segmentation

performance across the vast majority of test cases, rather than achieving high average scores only on easy cases. The reduction in low-score outliers further verifies that the proposed modules effectively mitigate the performance degradation caused by challenging cases with low-contrast boundaries, small lesion sizes, and irregular tumor shapes. These statistical observations are consistent with the quantitative results reported in the main manuscript, and provide additional evidence for the superior generalization and stability of GMANet over the baseline model.

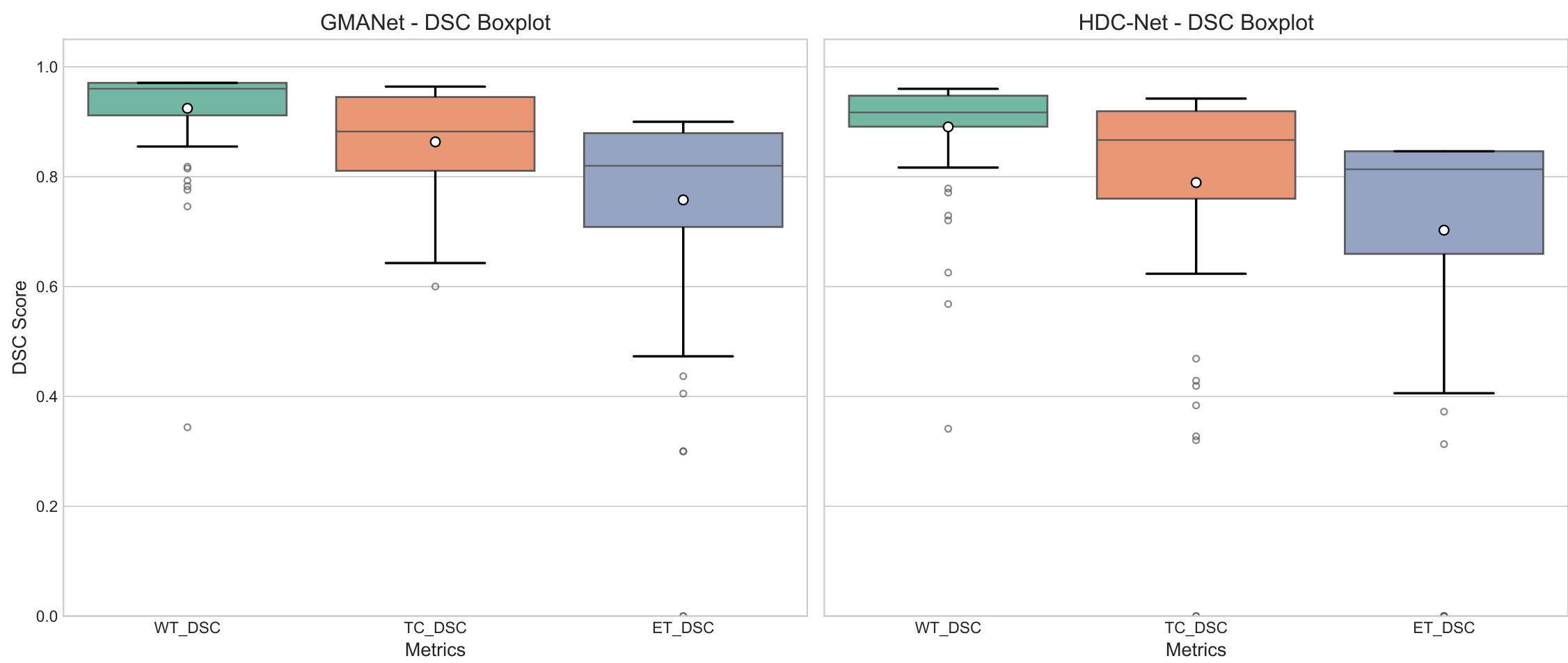


**Figure S1.** The results of DSC box plot

## 1.8 Comparison between GMANet and DPEA-Net under CPU-only condition

To further quantify the performance gap between the two models under the CPU-only deployment scenario that defines GMANet's clinical value, and to explicitly position this work relative to our prior publication, we provide a head-to-head comparison of GMANet and DPEA-Net on the BraTS2019 dataset, as summarized in Table S6.

Recently, we published DPEA-Net, a lightweight 3D CNN for glioma segmentation that achieves high ET Dice with only 0.17 M parameters. DPEA-Net was explicitly optimized for GPU-accelerated radiotherapy planning, minimizing GPU FLOPs and requiring a Tesla P100 GPU to reach practical inference speeds. On a standard CPU, DPEA-Net takes 36.92 seconds per volume — too slow for time-sensitive triage.

GMANet is not an iterative improvement of DPEA-Net, nor does it compete with it. Instead, GMANet answers a different research question: How can we achieve glioma segmentation in under 10 seconds on a CPU without GPU acceleration? As shown in the table, the two models differ fundamentally in four key dimensions:

- **Hardware target**: GPU (DPEA-Net) vs. CPU (GMANet)
- **Optimization objective**: Minimize GPU FLOPs (DPEA-Net) vs. minimize CPU latency (GMANet)
- **Clinical use case**: Radiotherapy planning & precision assessment (DPEA-Net) vs. initial triage & urgent referral (GMANet)
- **Key architectural modules**: DHDC + CDRSEA (DPEA-Net) vs. GMSD + FDA + LTU (GMANet)

Despite a minor increase in parameters and GFLOPs, GMANet delivers a 4.35× speedup in CPU inference time compared to DPEA-Net, while achieving higher WT Dice and TC Dice scores for the whole-tumor

and tumor core regions that are critical for rapid triage, with only a minor, clinically acceptable reduction in ET Dice that remains well above the 75% threshold for initial glioma screening.

These results confirm that the two models are optimized for non-overlapping use cases: DPEA-Net is designed for GPU-accelerated high-precision radiotherapy planning, while GMANet is purpose-built for fast, reliable CPU-only glioma triage in resource-limited settings, with no redundant functional overlap between the two. The two models are strictly complementary and form a two-stage clinical pathway: GMANet enables rapid on-site screening in primary care, emergency, or intraoperative MRI suites; DPEA-Net then provides high-precision segmentation at tertiary centers for definitive treatment planning. This manuscript focuses solely on the former, previously unmet need.

**Table S6.** Comparative analysis between DPEA-Net and GMANet across various dimensions.

| Dimension | Metric | DPEA-Net | GMANet (this work) |
|---|---|---|---|
| Model Parameters | Parameters | 0.17M | 0.20M |
| | GFLOPs | 17.48 | 20.10 |
| Inference Performance | CPU Time (s) | 36.92 s | 8.49 s (4.35× faster) |
| | Hardware Requirement | GPU (Tesla P100) | CPU (any laptop/server) |
| | Optimization Target | Minimize GPU FLOPs (17.48G) | Minimize CPU latency ($<$10 s) |
| Segmentation Accuracy | WT Dice (BraTS2019) | 90.43% | 92.45% |
| | TC Dice (BraTS2019) | 85.56% | 86.28% |
| | ET Dice (BraTS2019) | 81.89% | 78.42% (still $\geq$75% triage threshold) |
| Clinical Application | Primary / Target Use | Radiotherapy planning | Rapid triage & referral decision |

## 1.9 Clinical Utility and Failure Mode Analysis of GMANet

The volume estimation error, referral simulation, and ET Dice stratified by tumor size were derived from a representative run of GMANet, while the subtype analysis was based on the final GMANet model. Both runs exhibit consistent trends, and the key findings are reproducible across model variants. This section provides detailed results of the clinical utility and failure mode analysis of GMANet based on the BraTS2019 test set, including ET volume estimation error, referral simulation, ET Dice stratified by tumor size, false negative analysis, and performance comparison between HGG and LGG. All analyses were performed using the same test set partition as described in the main manuscript.

### Volume Estimation Error and Referral Simulation

For each test case, the ET volume was computed from both the predicted segmentation and the ground truth mask using isotropic voxel spacing of $1 \times 1 \times 1$ mm³. The absolute volume error was defined as $|V_{\text{pred}} - V_{\text{gt}}|$ and the relative volume error as $|V_{\text{pred}} - V_{\text{gt}}|/V_{\text{gt}} \times 100\%$. The median absolute ET volume error was 2.366 cm³, and the median relative volume error was 22.2%. Figure S2 shows the scatter plot of predicted vs. ground truth ET volumes, and Figure S3 presents the boxplot of relative volume error stratified by ground truth ET size groups.

To simulate a clinical referral decision, we used an ET volume threshold of 0.5 cm³ . Against the ground truth referral status, the confusion matrix was: True Positive = 58, False Positive = 2, True Negative = 3, False Negative = 5. Table S7 reports the derived performance metrics with 95% confidence intervals.

**Table S7.** Referral simulation performance (threshold = 0.5 cm³).

| Metric | Value | 95% CI lower | 95% CI upper |
|---|---|---|---|
| Sensitivity | 0.921 | 0.824 | 0.974 |
| Specificity | 0.600 | 0.147 | 0.947 |
| Positive Predictive Value (PPV) | 0.967 | 0.885 | 0.996 |
| Negative Predictive Value (NPV) | 0.375 | 0.085 | 0.755 |

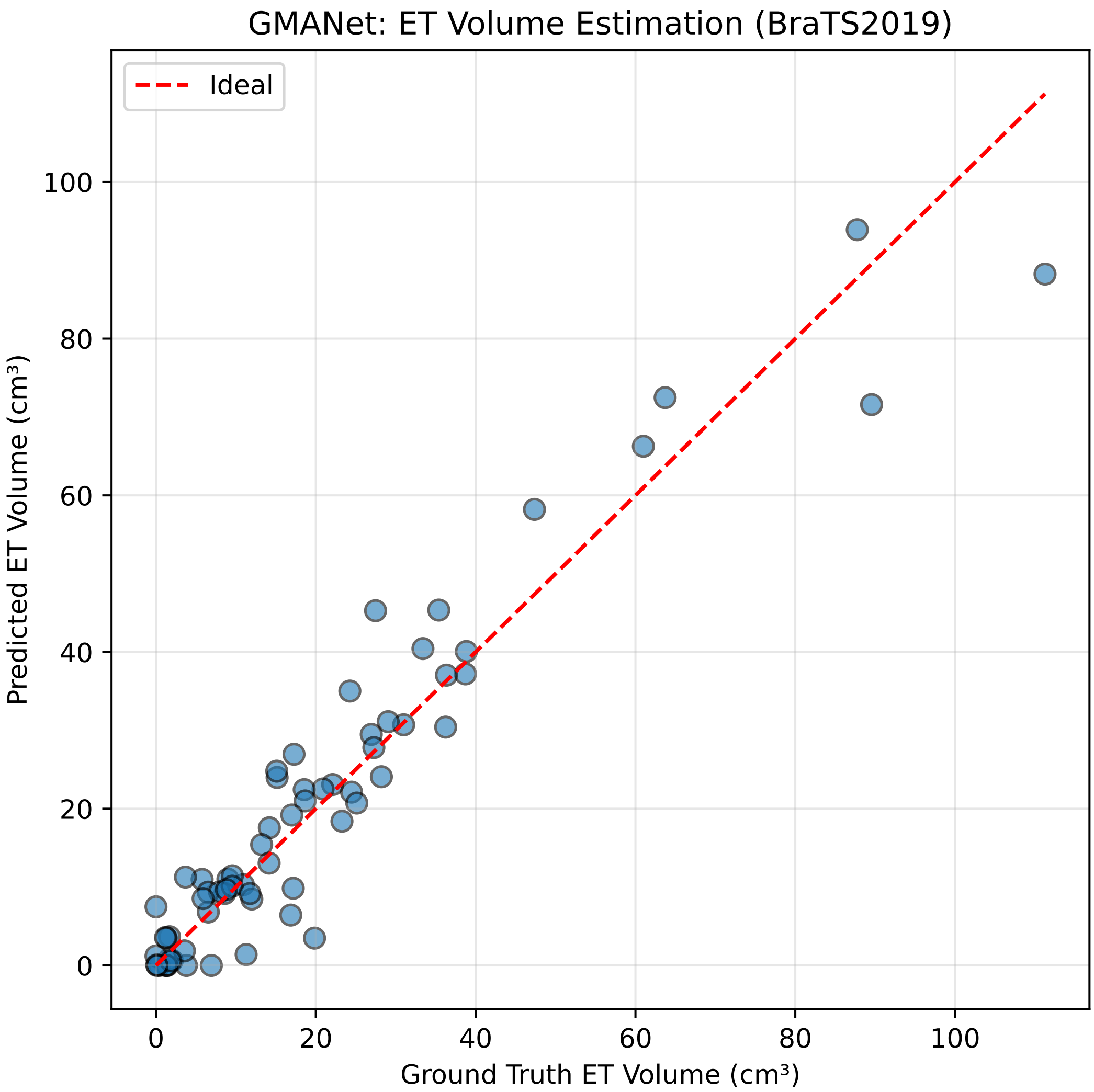


**Figure S2.** Scatter plot of predicted vs. ground truth ET volume.

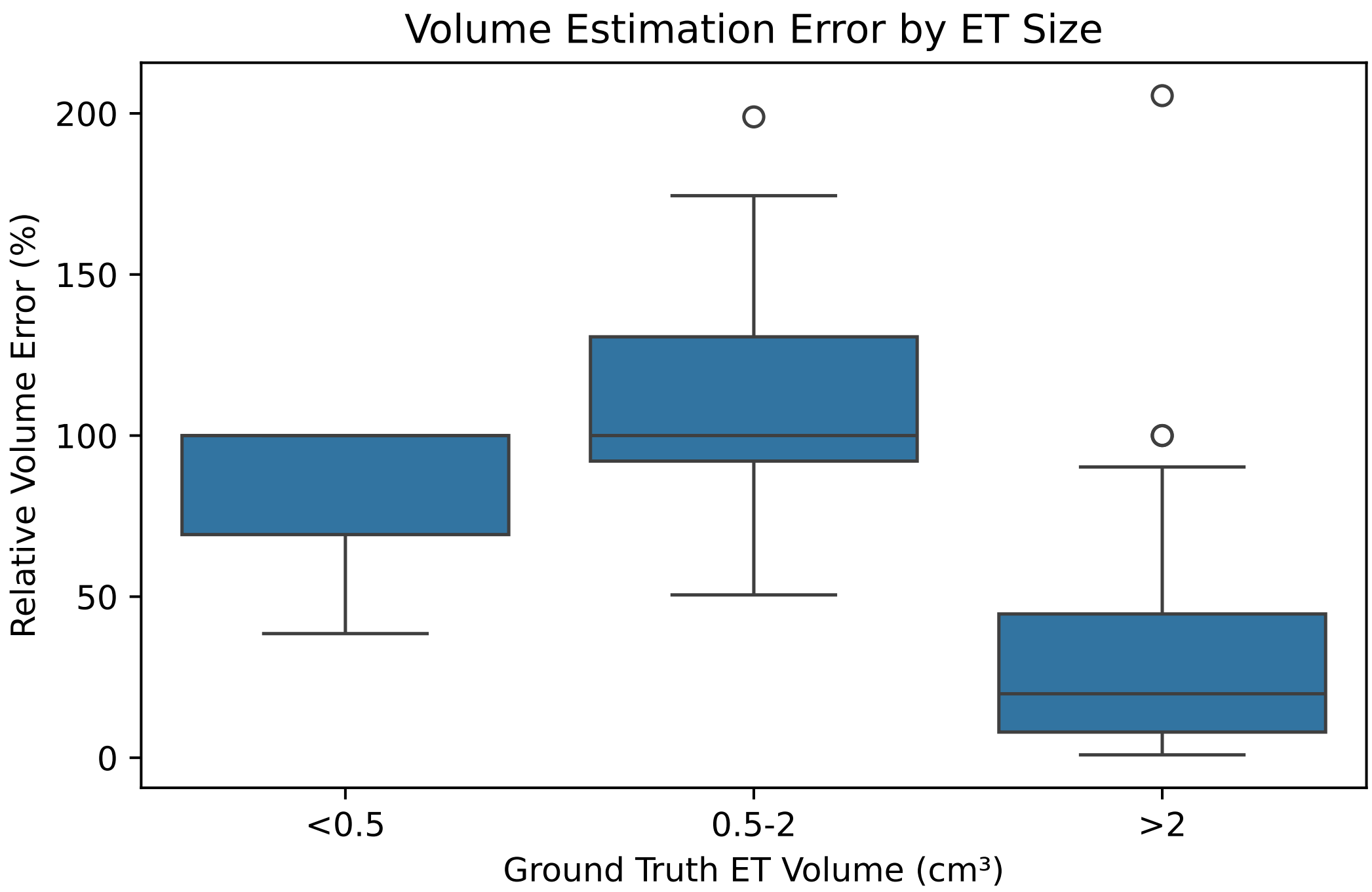


**Figure S3.** Relative volume error stratified by ground truth ET size groups.

## ET Dice Stratified by Tumor Size and False Negative Analysis

To investigate the dependence of ET segmentation quality on lesion size, we divided the test cases into three groups based on ground truth ET volume: $< 0.5$ cm³ , $0.5$–$2$ cm³ , and $> 2$ cm³ . The mean, standard deviation, median, and quartiles of ET Dice for each group are summarized in Table S8. Figure S4 shows the boxplot of ET Dice across the three size groups, and Figure S5 presents the scatter plot of ET Dice versus ground truth ET volume on a logarithmic scale, with a horizontal line at the clinically acceptable threshold of 0.75.

A false negative was defined as a clinically significant ET lesion for which the model predicted an ET volume of less than 0.1 cm³. The overall false negative rate was 7.9% . The five false negative case IDs are: `BraTS19_CBICA_ARZ_1`, `BraTS19_CBICA_AVB_1`, `BraTS19_TCIA02_283_1`, `BraTS19_TCIA10_330_1`, and `BraTS19_TCIA10_420_1`. Visual inspection of these cases revealed that the missed ET lesions were typically small, low-contrast, and located at the infiltrative tumor periphery, which are known challenging scenarios even for larger models.

**Table S8.** ET Dice stratified by ground truth ET volume.

| Size group | N | Mean (SD) | Median | IQR | Min–Max |
|---|---|---|---|---|---|
| $< 0.5$ cm³ | 3 | 0.178 (0.308) | 0.000 | 0.000–0.267 | 0.000–0.534 |
| $0.5$–$2$ cm³ | 8 | 0.312 (0.271) | 0.399 | 0.000–0.508 | 0.000–0.625 |
| $> 2$ cm³ | 54 | 0.732 (0.229) | 0.800 | 0.732–0.876 | 0.000–0.932 |

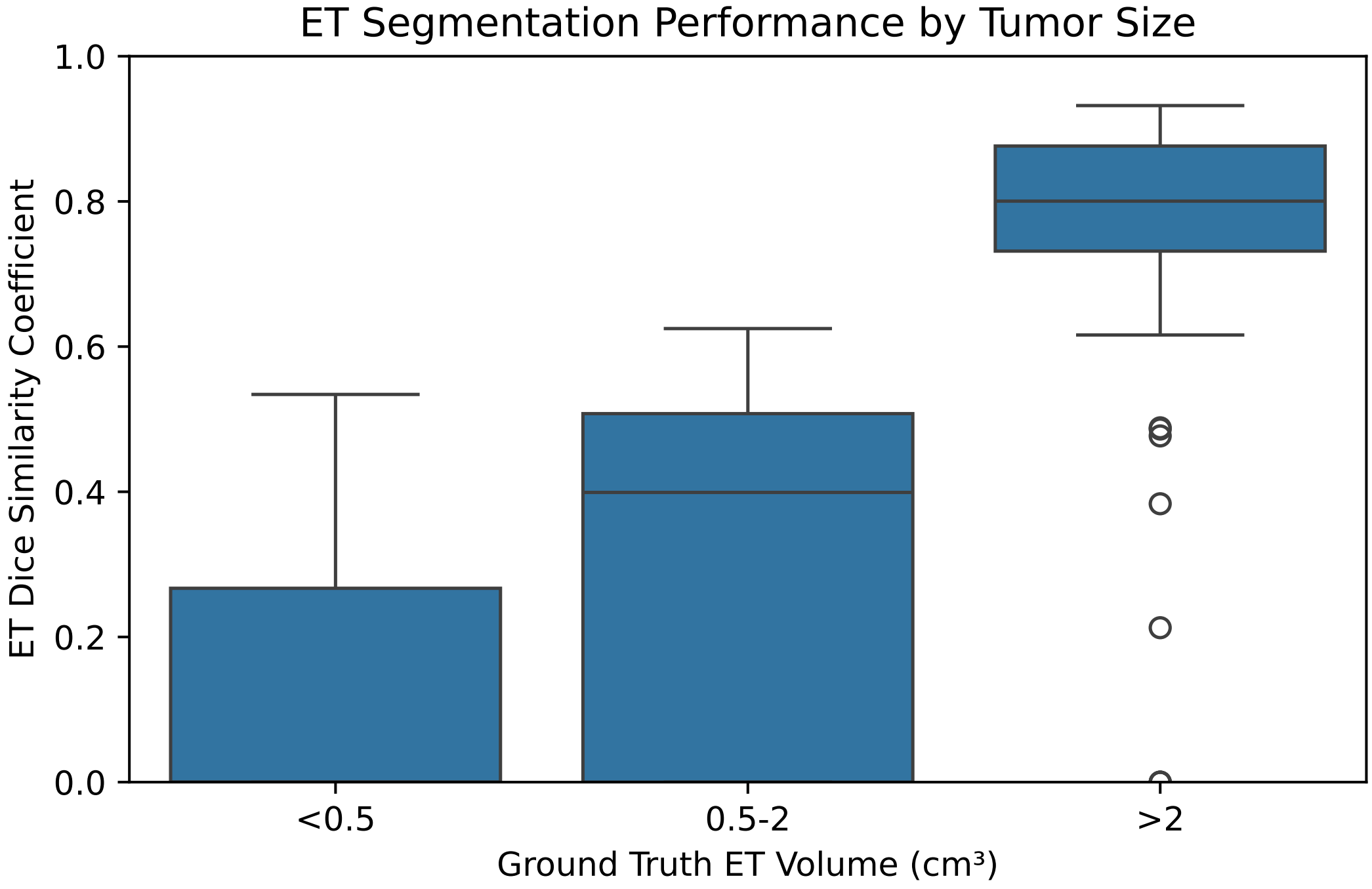


**Figure S4.** Boxplot of ET Dice stratified by ground truth ET size groups.

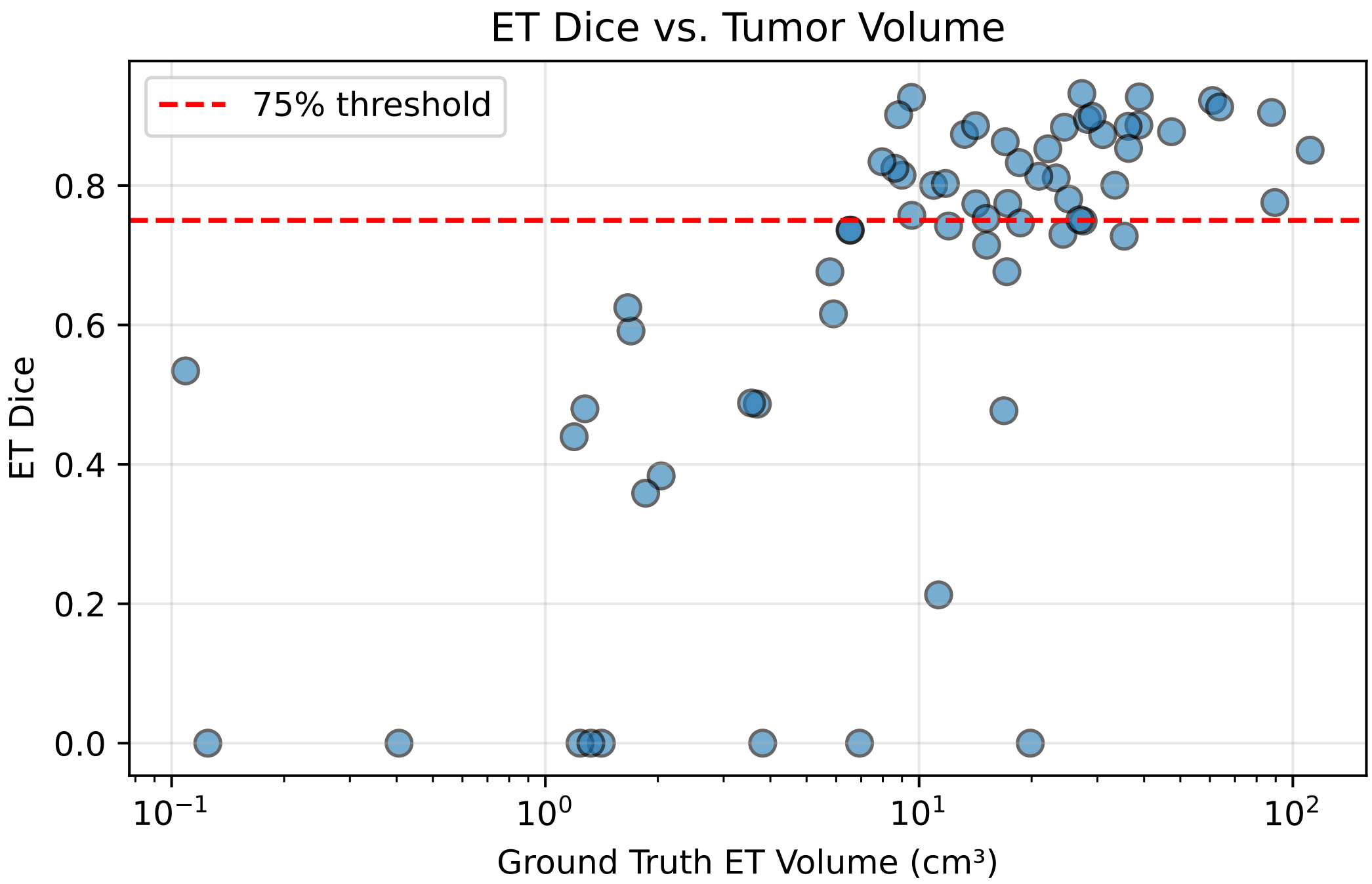


**Figure S5.** Scatter plot of ET Dice versus ground truth ET volume (log scale). The red dashed line marks the 75% threshold.

### Stratification by Tumor Subtype.

The test set was stratified into high-grade gliomas (HGG, n=52) and low-grade gliomas (LGG, n=16) based on the original BraTS annotations. Table S9 compares the Dice similarity coefficients for WT, TC, and ET between the two subtypes. For the ET region, HGG achieved a significantly higher median Dice than LGG, with a Mann–Whitney U test p-value of 0.036. This difference is clinically expected because LGG typically exhibits subtle or absent contrast enhancement, making ET segmentation inherently more difficult. No statistically significant differences were observed for WT or TC.

**Table S9.** Segmentation performance stratified by tumor subtype.

| Region | HGG median (IQR) | LGG median (IQR) | U statistic | p-value |
|---|---|---|---|---|
| WT | 0.950 (0.906–0.971) | 0.971 (0.950–0.971) | 305.0 | 0.095 |
| TC | 0.902 (0.841–0.949) | 0.868 (0.738–0.888) | 551.0 | 0.052 |
| ET | 0.827 (0.784–0.882) | 0.678 (0.582–0.855) | 561.5 | 0.036 |
